# X-crossing pneumatic artificial muscles

**DOI:** 10.1126/sciadv.adi7133

**Published:** 2023-09-20

**Authors:** Miao Feng, Dezhi Yang, Lei Ren, Guowu Wei, Guoying Gu

**Affiliations:** ^1^ Robotics Institute, School of Mechanical Engineering, Shanghai Jiao Tong University, Shanghai 200240, China.; ^2^ State Key Laboratory of Mechanical System and Vibration, Shanghai Jiao Tong University, Shanghai 200240, China.; ^3^ Department of Mechanical, Aerospace and Civil Engineering, The University of Manchester, Manchester M13 9PL, UK.; ^4^ School of Science, Engineering and Environment, The University of Salford, Salford M5 4WT, UK.; ^5^ Key Laboratory of Bionic Engineering, Jilin University, Changchun 130015, China.; ^6^ Meta Robotics Institute, Shanghai Jiao Tong University, Shanghai 200240, China.

## Abstract

Artificial muscles are promising in soft exoskeletons, locomotion robots, and operation machines. However, their performance in contraction ratio, output force, and dynamic response is often imbalanced and limited by materials, structures, or actuation principles. We present lightweight, high–contraction ratio, high–output force, and positive pressure–driven X-crossing pneumatic artificial muscles (X-PAMs). Unlike PAMs, our X-PAMs harness the X-crossing mechanism to directly convert linear motion along the actuator axis, achieving an unprecedented 92.9% contraction ratio and an output force of 207.9 Newtons per kilogram per kilopascal with excellent dynamic properties, such as strain rate (1603.0% per second), specific power (5.7 kilowatts per kilogram), and work density (842.9 kilojoules per meter cubed). These properties can overcome the slow actuation of conventional PAMs, providing robotic elbow, jumping robot, and lightweight gripper with fast, powerful performance. The robust design of X-PAMs withstands extreme environments, including high-temperature, underwater, and long-duration actuation, while being scalable to parallel, asymmetric, and ring-shaped configurations for potential applications.

## Teaser

Positive pressure-driven pneumatic artificial muscles achieve an unprecedented contraction ratio and comprehensive performance.

## INTRODUCTION

Artificial muscles are emerging as a key actuation component of soft machines and robots, owing to their flexibility, safety, and intelligence. To achieve competitive mechanical performance with skeletal muscles (table S1) (*1*–*6*), scientists have invented various artificial muscles based on (i) new materials, such as hydrogel (*7*, *8*), carbon nanotubes (*9*, *10*), piezo materials (*11*, *12*), ionic polymer-metal composites (*11*), dielectric/liquid crystal elastomers (*13*–*16*), shape memory alloys/polymers (*17*, *18*), and conductive/ferroelectric polymers (*2*, *4*, *19*, *20*); (ii) specialized structures, such as supercoiled polymer actuators (*21*, *22*), twisted string actuators (*23*, *24*), and hydraulically amplified self-healing electrostatic actuators (*3*, *25*); or (iii) versatile stimuli, such as fluidic (*23*, *26*–*28*), electric (*29*, *30*), thermal (*16*, *22*), light (*7*, *8*), chemical (*31*, *32*), humid (*21*), and hybrid energy sources (table S2) (*33*). Although these developments show advantages in some aspects, the design of artificial muscles with simple mechanisms and excellent comprehensive mechanical performance, including contraction ratio, output force, and dynamic properties, remains elusive.

Pneumatic artificial muscles (PAMs), the most prevailing fluidic actuators, are promising due to their potential in simplicity, portability, safety, economy, and mechanical properties (*26*, *34*, *35*). Among them, vacuum-based PAMs (V-PAMs) achieve a state-of-the-art contraction ratio of 90% (99.7% with rigid components) (*26*, *36*). However, a negative pressure is necessary for these designs, which can only supply a maximum differential pressure of 1 atm. This limitation restricts the mechanical performance of V-PAMs, which, in contrast, is not an issue for the positive pressure–driven PAMs. For example, McKibben artificial muscles, invented in the 1950s, can offer considerable output force (*34*, *37*). Nevertheless, their theoretical maximum contraction ratio is only 36.4%, and they typically require high pressure for high output force, which is usually inefficient (*34*, *37*, *38*).

To address these challenges in positive pressure–driven PAMs, researchers have proposed several variants of McKibben muscles by pleating the air chamber (*35*, *39*), optimizing the radial expansion (*38*, *40*, *41*), and introducing external constraints (*42*), which achieve an optimal contraction ratio of 71% with partial mechanical performance improved. In addition, Cavatappi artificial muscles, composed of coiling tubes, and modular multichamber artificial muscles that adopt rigid pathing shells are also developed for portability or configuration advantages (*23*, *43*). However, these positive pressure–driven PAMs share the same actuation principle of “converting radial expansion into axial contraction” or are enabled by complicated mechanisms.

**Fig. 1.**
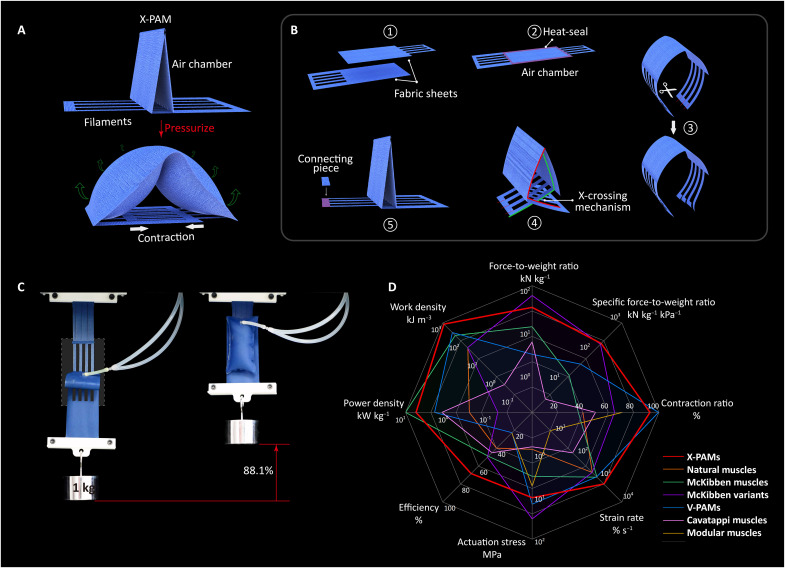
X-crossing pneumatic artificial muscles. (**A**) Actuation principle of X-crossing PAMs (X-PAMs). When pressurized, X-PAMs transfer the expansion of the air chamber to the contraction of the filaments along the actuator axis. (**B**) Structure of X-PAMs. Two fabric sheets fabricate an X-PAM by folding the actuator and crossing the filaments of both sides into the X-crossing mechanism. (**C**) A typical X-PAM (i.e., the type 2 in the following text) lifts a load of 1 kg with a maximum contraction ratio of 88.1% under 100 kPa. (**D**) Performance indicators of X-PAMs and the comparison with various PAMs, which are McKibben (McKibben artificial muscles), McKibben variants (variants of McKibben artificial muscles), V-PAMs, Cavatappi (Cavatappi artificial muscles), and modular multichamber (pneumatic artificial muscles based on modular multichamber soft actuator). These data come from table S1.

In this work, we present the concept of “directly transmitting linear motion along the actuator axis” and design X-crossing PAMs (termed X-PAMs) driven by positive pressure (Fig. 1, A to C). The X-PAMs, composed of entirely soft fabric, achieve an unprecedented contraction ratio of 92.9% and an excellent comprehensive mechanical performance in strain rate, actuation stress, specific power, work density, force-to-weight ratio, and efficiency (table S1 and Fig. 1D). Meanwhile, a series of experiments demonstrates their potential in various soft robotic applications, extreme environments, and mechanical extendability.

## RESULTS

### The design and actuation principle of X-PAMs

The design and actuation principle of X-PAMs are illustrated in Fig. 1 (A to C). Typically, an X-PAM comprises two flexible fabric sheets of woven nylon with thermoplastic polyurethane (TPU) coating (fig. S1, movie S1, and Supplementary Texts). Each sheet features a rectangular area for pressurization and a comb-like area working as “filaments” to transmit force and motion. These rectangular areas are heat-sealed together at 220°C to form an airtight chamber (*44*), while the filaments are further crossed to create the X-crossing mechanism by folding the chamber (fig. S2 and movie S2). When depressurized, the X-PAM is mechanically transparent due to the bending flexibility of the air chamber. When pressurized, the inflatable chamber rapidly stiffens, resists bending, and expands straight. Harnessing the unique design and actuation principle, the X-crossing mechanism directly transmits the chamber expansion into linear contraction along the actuator axis and can achieve excellent comprehensive performance (Fig. 1D).

We should note that the fabric is anisotropic, and the anisotropy can benefit the actuation performance of X-PAMs (fig. S1). Here, the fabric direction of higher stiffness is aligned with the filament direction of X-PAMs to reduce the axis strain of the material and improve its breaking strength. Accordingly, the fabric direction of lower stiffness is along the cross-sectional circumference of the chamber, which helps the chamber inflate with a larger section area and enhance the output force.

### Force-displacement relations of X-PAMs

**Fig. 2.**
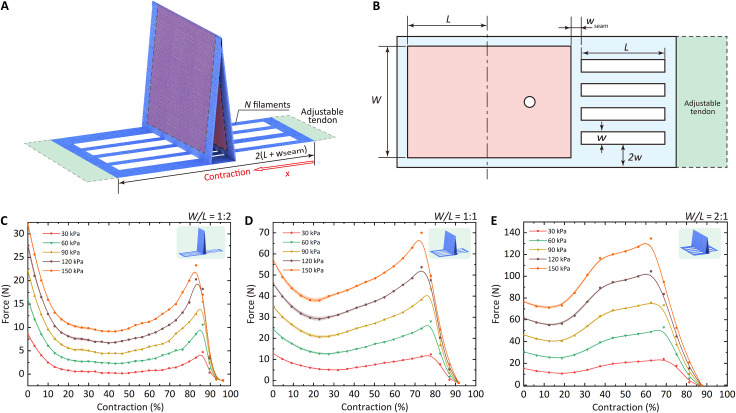
Geometry and static characterization of X-PAMs. (**A**) Three-dimensional (3D) structure of X-PAMs. (**B**) Parameters of the upper 2D fabric sheet of the X-PAM. For the lower sheet; see fig. S2A. (**C**) Output force of the X-PAM (type 1, *W*/*L* = 1:2). (**D**) Output force of the X-PAM (type 2, *W*/*L* = 1:1). (**E**) Output force of the X-PAM (type 3, *W*/*L* = 2:1). The chamber area (red shadow) of all these three exemplary X-PAMs is maintained at 5000 mm^2^. All these presented deviations or error bars are SDs (±SD) in this work.

We next parameterize the geometry of the X-PAMs and characterize their force-displacement relations (Fig. 2, fig. S2A, and Supplementary Texts). The primary geometric parameters of the X-PAMs are *L* (the length of the chamber when folded), *W* (the width of the air chamber), *N* (the number of filaments), *w*_seam_ (the polytechnical width of the seam), and *w* (the width of each filament).

The contraction ratio is the ratio (*38*, *43*, *45*) of the contraction displacement *x* to the active length

$$\text{Contraction ratio}=\frac{x}{2(L+w_{\mathrm{seam}})}\times 100\%$$

We measure the force-displacement relations of the three types of X-PAMs from 0 to 150 kPa, which share the same chamber area of 5000 mm^2^ but different aspect ratios *W*/*L*, i.e., type 1 (*W*/*L* = 1:2), type 2 (*W*/*L* = 1:1), and type 3 (*W*/*L* = 2:1) (see Supplementary Texts).

Experimental results show that the output force of these three types of X-PAMs remains at a high level over a wide range of contraction ratios on the whole and successively experiences declining, rising, and declining (Fig. 2, C to E), in accordance with the trend presented in the simplified model (fig. S3 and Supplementary Texts). This trend is because the output force is dominated by chamber squeezing in the early contraction stage. With the expansion of the chamber in the following stage, the chamber configuration (i.e., the bending angle of the chamber) gradually becomes the dominating factor in the output force. When the output force drops to zero, these X-PAMs reach their maximum contraction ratios of 92.9, 91.6, and 87.6%, respectively.

Furthermore, experiments and simulations demonstrate that the output force of these three X-PAMs increases with input pressures at each position (Fig. 2, C to E; fig. S4; and Supplementary Texts). Their local maximum output forces under 150 kPa are 23.3 ± 0.2 N (83.0%), 70.0 ± 0.1 N (73.2%), and 134.7 ± 0.8 N (62.6%), respectively. The corresponding force-to-weight ratios are 5.2 ± 0.0 kN kg^−1^, 16.0 ± 0.0 kN kg^−1^, and 31.2 ± 0.2 kN kg^−1^, mapping to the specific force-to-weight ratios of 34.8 ± 0.3 N kg^−1^ kPa^−1^, 106.6 ± 0.2 N kg^−1^ kPa^−1^, and 207.9 ± 1.3 N kg^−1^ kPa^−1^, respectively, which is prominent among existing pneumatic actuators (Fig. 1D and table S1).

### Dynamic properties of X-PAMs

**Fig. 3.**
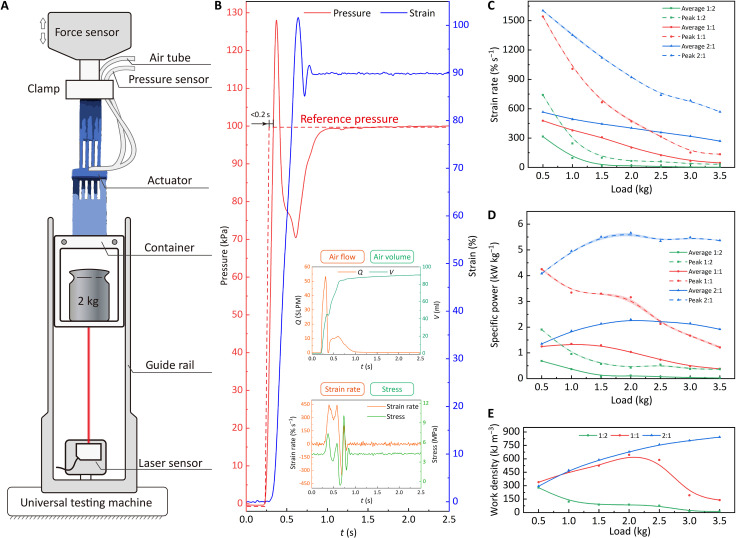
Step response and actuation performance of X-PAMs. (**A**) Experimental setup. The X-PAM is connected to an air tube and pressure sensor. Here, displacement, force, mass flow, and air pressure are recorded. The mass of the load is 0.5:0.5:3.5 kg with the container included. (**B**) Pressure, strain, air flow, chamber volume, strain rate, and actuation stress of the X-PAMs actuated by step stimuli, of which the actual pressure reaches the expected value in no more than 0.2 s. SLPM, standard liters per minute. (**C**) Average and peak strain rate of X-PAMs. (**D**) Average and peak specific power of X-PAMs. (**E**) Work density of X-PAMs.

To investigate the dynamic responses of X-PAMs, we perform the tests under a uniform step pressure of 100 kPa (thus avoiding the influence of air supply factors) with loads of 0.5:0.5:3.5 kg applied (Fig. 3A, fig. S5, and Supplementary Texts). When the step pressure is stimulated, the pressure in the air chamber instantly (<0.2 s) rises to 100 kPa, ensuring that the X-PAM nearly has no time to start contracting. With compressed air flowing into the chamber, the X-PAM expands and lifts its load (Fig. 3B and movie S3).

Experimental results show that primary indicators for dynamic responses of X-PAMs are working condition–dependent (Fig. 3, C to E; fig. S6, A and B; and table S1). With the load increase, the strain rate decreases, and the actuation stress increases. The maximum average and peak strain rates of the type 3 X-PAM can be 565.5 ± 2.9% s^−1^ and 1603.0 ± 7.1% s^−1^. For energy indicators, i.e., specific power, work density, and efficiency, the X-PAMs can maintain them at a high level unless the load is too heavy to be lifted with enough displacement (e.g., partial data of type 1 and 2 X-PAM). The maximum specific power, work density, and efficiency of the type 3 X-PAM are 2.3 ± 0.0 kW kg^−1^ (average) and 5.7 ± 0.1 kW kg^−1^ (peak), 842.9 ± 1.6 kJ m^−3^, and 68.3 ± 0.7%, respectively. These results demonstrate that a large geometry aspect of *W*/*L* can benefit the dynamic response of the X-PAM. For a specific application, we can obtain excellent comprehensive mechanical performance when the X-PAM is designed with the load requirements. Besides, the force and displacement tracking are also executed (movie S4).

### Performance and ability in typical soft robotic applications

**Fig. 4.**
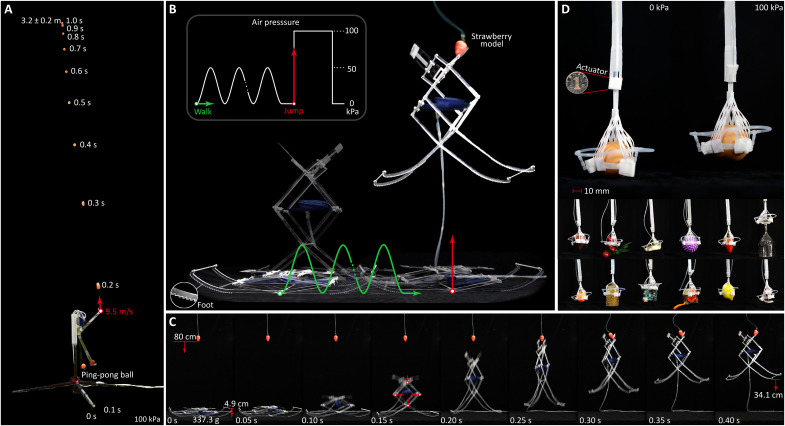
Typical soft robotic applications of X-PAMs. (**A**) Robotic elbow. X-PAMs can be used as an artificial muscle in exoskeletons, e.g., a robotic elbow, where a ping-pong ball is vertically thrown to a height of 3.2 ± 0.2 m with an initial speed of 9.5 m s^−1^. (**B**) Jumping robot. X-PAMs endow a robot (337.3 g) with the capability of faster crawling and higher jumping to 6.9 times (34.1 ± 4.5 cm) its relaxed body height (4.9 cm). (**C**) Details of the jumping process. (**D**) Soft gripper (1.9 g). Four small-scale X-PAMs are combined as a lightweight gripper to grasp eggs, cups, fruits, and electric boards with a force-to-weight ratio of 45.4.

To explore the abilities of X-PAMs in soft robotic applications, we first integrate an X-PAM into a real-size robotic elbow (Fig. 4A; fig. S7, A to C; movie S5; and Supplementary Texts), where a geometrically comparable McKibben muscle with the same initial length and circumference as the X-PAM is enrolled for comparisons in the experiments. Under 100 kPa, the robotic elbow with X-PAM can flex more than 125° and stably withstand a weight of 1 kg, while the load-free McKibben muscle only actuates the robotic elbow to less than 60°. For a step stimulus of 100 kPa, the robotic elbow with X-PAM vertically throws a ping-pong ball (2.8 g) to a height of 3.2 ± 0.2 m (with an initial speed of about 9.5 m s^−1^), which is dozens of times the height achieved by its pneumatic and electrical pioneers of artificial muscles (*23*, *29*). In contrast, the traditional McKibben muscle even could not throw the ping-pong ball off the robotic elbow. These experiments show that X-PAMs have a large contraction ratio, a considerable output force, and a high strain rate.

For the performance of X-PAMs in more complicated soft machines, we develop a jumping robot with a “diamond” mechanism (337.3 g; Fig. 4, B and C; fig. S7D; movie S6; and Supplementary Texts) actuated by the X-PAM. The jumping robot is stimulated by a sinusoidal pressure (0 to 50 kPa, 0.1 Hz) to walk 60 cm and, then, under a step pressure of 100 kPa, jumps up to “eat” the plastic strawberry model hung to 80 cm. Experiments show that the jumping robot with X-PAM (type 3) has a more considerable step length (10.0 cm per step) and walks faster (2.5 cm s^−1^) than that with the McKibben muscle (1.1 cm per step, 0.1 cm s^−1^). Meanwhile, the jumping robot actuated by the X-PAM is able to successfully eat the strawberry model with its foot off the ground by 6.9 times (34.1 ± 4.5 cm) of its relaxed body height (4.9 cm), which is a remarkable achievement for jumping robots (*46*, *47*), especially pneumatic robotic machines. Such walking and jumping ability validates the large contraction ratio and high strain rate, further unveiling its high power and work density.

Besides these applications actuated by one X-PAM, a group of X-PAMs can also comprise fully soft machines. We used four small coupled X-PAMs to fabricate a gripper (1.9 g), of which three X-PAMs perform the enveloping motion, and the other works as a lifter (Fig. 4D, fig. S7E, movie S7, and Supplementary Texts). This gripper can grasp an egg (56.9 g, diameter of 43.5 mm) to a height of 18.5 mm under 100 kPa. Moreover, it is effective for various objects, such as ping-pong balls, measuring tape, and microcontrollers, having a maximum load of 45.4 times its weight. This lightweight gripper demonstrates excellent scalability and integrability of X-PAMs.

### Potential applications in extreme environments

**Fig. 5.**
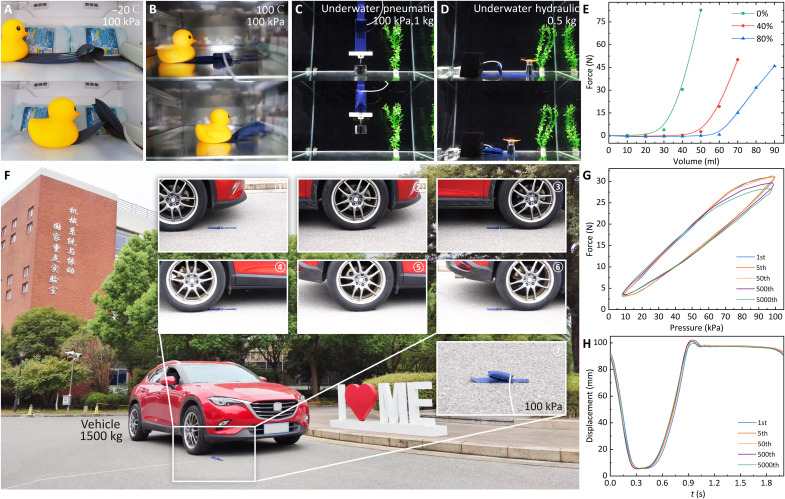
X-PAMs work in various extreme environments. A manually operated pressure of 100 kPa is applied, and all the tests adopt the same actuator, i.e., (**A**) to (**D**) and (**F**) using the same type 2 X-PAM (*W*/*L* = 1:1). (A) Actuation in a cold environment −20°C (after >5 min). (B) Actuation in a high temperature of 100°C (after >5 min). (C) Underwater pneumatic actuation with a load of 1 kg. (D) Dragging a load of 0.5 kg with underwater hydraulic actuation. (**E**) Output force of X-PAMs when hydraulically actuated. (F) A vehicle of about 1500 kg drives over the X-PAM, and the X-PAM can work well. (**G**) Repetitive output force in the 1st, 5th, 50th, 500th, and 5000th cycles (the contraction ratio is fixed at 50%). (**H**) Repetitive displacement in the 1st, 5th, 50th, 500th, and 5000th cycles (a load of 2 kg is applied). In (G) and (H), the type 2 X-PAM (*W*/*L* = 1:1) with a sinusoidal pressure (0 to 100 kPa, 0.5 Hz) is used.

Applications in various extreme environments are critical for practical artificial muscles, and we examine this ability of X-PAMs with the same actuator (Fig. 5, movie S8, and Supplementary Texts). We first put the X-PAM (type 2) in a refrigerator with a low temperature of −20°C and a thermostat with a high temperature of 100°C (Fig. 5, A and B). After no less than 5 min, the X-PAM normally contracts (100 kPa, the same pressure for the following pneumatic experiments). We proceed to immerse the X-PAM underwater and test its functionality using two distinct working mediums: compressed air and water. We observe that the X-PAM can effectively lift or drag the loads like working in the atmosphere (Fig. 5, C to E). Following the tests, the X-PAM is dried using a blast and then subjected to a rolling test, where a 1500-kg vehicle drives over it at 5 to 10 km hour^−1^ (Fig. 5F). The X-PAM maintains its exceptional air tightness and working ability, whether deflated or inflated when driven over (movie S8).

To further evaluate the performance under long-term working conditions, we conduct force and displacement durability tests on our X-PAMs (Fig. 5, G and H, and Supplementary Texts). Specifically, we conduct a force durability test on the type 2 X-PAM with more than 5000 cycles of operation (0 to 100 kPa, 0.5 Hz, fixed at the contraction ratio of 50%). For the displacement durability test, there is some surface abrasion on the fabric filaments after 5000 cycles; however, the X-PAM continues to function reliably with a load of 2 kg (0 to 100 kPa, 0.5 Hz). These results indicate that our X-PAMs can deliver sustained performance under demanding operational conditions.

### Extendibility of X-PAMs for soft robots

**Fig. 6.**
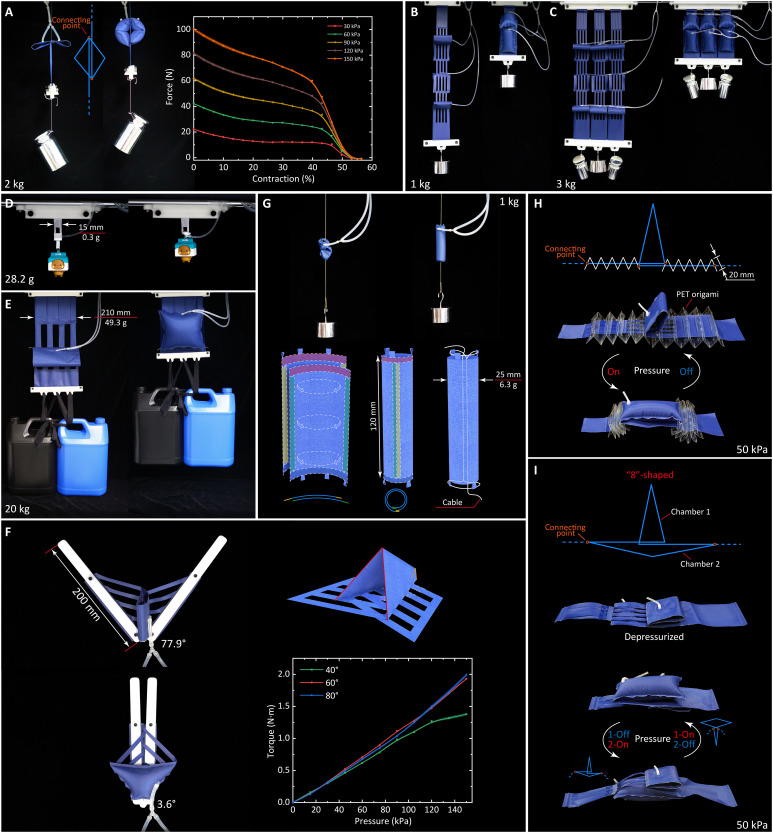
Extensive actuators with X-crossing mechanism. Unless otherwise specified, the air pressure below is 100 kPa. (**A**) X-PAM couple (41.5%), achieving higher output force than a single X-PAM. (**B**) Serial X-PAMs (88.8%), achieving a larger displacement. (**C**) Parallel X-PAMs (91.0%), enhancing the output force while keeping large displacement. (**D**) X-PAMs are scalable to be lightweight with small-scale sizes (93.8%). (**E**) Large-scale X-PAMs can lift a heavy load (50 kPa, 72.3%). (**F**) The air chamber (also the filaments) of X-PAMs can morph into designed shapes, such as trapezoids with nonuniform output force and displacement. Here presents an asymmetric X-PAM actuating a scissor mechanism. (**G**) The original air chamber is not limited to a planar. Here is a ring-shaped X-PAM that morphically looks like mammal muscle (33.8%). (**H**) Origami-combined X-PAM simultaneously achieves superior mechanical transparency and resilience (between 11.7 and 85.1%, 50 kPa, 0.2 Hz). PET, polyethylene terephthalate. (**I**) The digit 8-shaped X-PAM with two chambers can achieve active and powerful contraction and extension (between 0 and 91.6%, 50 kPa, 0.1 Hz).

The experiments above demonstrate the properties and performance of the original X-PAM (Fig. 1C). However, the potential of X-PAMs and our design concept of directly transmitting linear motion along the actuator axis can extend beyond one unit or configuration (see details in movie S9 and Supplementary Texts). We can design an “X-PAM couple” to enhance the output force by symmetrically connecting two actuators (Fig. 6A). We can also cascade a group of actuators for serial X-PAMs with high displacement (Fig. 6B). On the other hand, we demonstrate that parallel X-PAMs can further improve the output force (Fig. 6C). As illustrated in the soft gripper application, X-PAMs are scalable, not only to a small-size actuator with lightweight performance (Fig. 6D) but also to a large-size one with strong carrying capacity (Fig. 6E). Besides, the asymmetric X-PAM with a trapezoid chamber can produce rotation motion to actuate some rotating mechanisms, such as robotic scissor, with considerable output torque (Fig. 6F and fig. S8A). Further morphing the two-dimensional (2D) chamber onto a 3D cylindrical surface can produce a muscle-like ring-shaped X-PAM (Fig. 6G). Note that our fabric-based X-PAMs have better mechanical transparency than most silicone-based or resin-based PAMs. However, mechanical resilience for extension might be required in some applications, which could not be an issue by combining origami with the X-PAM to balance these properties (Fig. 6H). Moreover, this extension can be active and powerful by integrating two X-PAM chambers into the digit “8” shape (Fig. 6I). All these prototypes demonstrate the excellent extendibility and scalability of our X-PAMs for various soft robots.

## DISCUSSION

This work presents the positive pressure–driven X-PAMs based on the X-crossing mechanism that enables parallelly relative sliding between filaments, like the sliding myosin and actin myofilaments in skeletal muscles (*48*, *49*). Such X-PAMs directly transmit linear motion along the actuator axis and achieve a high contraction ratio of 92.9% in the exemplary actuators, whose theoretical limit is 100%. Unlike most PAMs with monotonically and sharply decreasing output force (*37*, *39*, *40*), our X-PAMs can maintain a high output force for a wide range of contraction ratios due to the chamber squeezing and configuration evolving. This unique feature may grant X-PAMs excellent properties, including strain rate, specific power, and work density, making them suitable for fast and powerful applications, such as jumping robots. Furthermore, X-PAMs are highly extendable and can function well under extreme environmental conditions.

For a given pressure stimulation, X-PAMs can perform faster and more powerfully than state-of-the-art PAMs in transient dynamic tasks. Regarding continuous dynamic responses (fig. S9), X-PAMs can also accurately track a desired force, and the cutoff frequency of force response is considerable. Comparatively, because the output force of X-PAMs is high and nonlinear with the wide contraction span, the displacement controllability of X-PAMs is not outstanding, which might be improved by introducing elastic components and tuning the force-displacement relationship. Meanwhile, the bandwidth of displacement response might benefit from optimizing air consumption, improving the speed of air exhaust, and incorporating the air recirculation mechanism according to specific application requirements (*28*).

Among existing works, the pressurized chamber of most contracting PAMs expands in all directions, which may interfere with other mechanical components, e.g., an actuator against the robotic upper arm. Our X-PAMs, expanding unilaterally, have the advantage of partially mitigating this issue. However, we should mention that the longitudinal chamber expansion of X-PAMs still exists and might impede the contraction for some applications where the adjustable tendons are required to be very short due to space restriction. Besides, our displacement durability test highlights the importance of using wear-resistant and frictionless materials to minimize friction between rough surfaces and improve performance in long-duration function and displacement controllability.

We anticipate that the design concept of directly transmitting linear motion along the actuator axis will inspire further research in artificial muscles. Future works include investigations into novel materials, such as carbon nanotubes, diverse structures like origami, and various stimuli including magnetic and even their hybrid, to achieve superior mechanical performance and versatile applications, covering the microcosm and macrocosm.

## MATERIALS AND METHODS

### Materials and fabrication of X-PAMs

Primary materials (figs. S1 and S2 and Supplementary Texts) comprising X-PAMs are N210D rib weft-knitted polyester fabric with TPU coating ($3.0/m^2^, 0.2 mm; Jiaxing Yingcheng Textile Co. Ltd., China), TPU air connecter ($0.11/piece, Φ3; Shenzhen Jianda Handbag Accessories Co. Ltd., China), and silicone tubes (Φ4; Daoguan Co. Ltd., China). An optic microscope (SN-300; SangNond, China) imaged the textile. For the small-scale X-PAM, the textile is Nylon N66 fabric with TPU coating. The comparative McKibben muscles are made of commercial balloons (maximum diameter 45.7 cm when inflated; Chongqing Junhe Trading Co. Ltd., China), plastic chambers (tube polyethylene membrane with thickness of 0.06 mm, folded width 90 mm; Yu Mei Co. Ltd., China), nylon woven network sheath (width of 25 and 45 mm; Shenzhen Xinhong Electronic Materials Co. Ltd., China), fabric sheaths (tube cotton woven textiles, folded width of 70 mm; Yunbu Workshop, China), and polyurethane tubes (Φ4; Taiwan Shannaisi Pneumatic International Group, China).

A set of tools is used to fabricate X-PAMs, including an acrylic bending machine (type A; Jinan Hongyang CNC Machinery Co. Ltd., China), a laser cutter (VLS 3.50; Universal Laser Systems, USA), and cyanoacrylate adhesive (Xunlei Co. Ltd., China). The fabrication of X-PAMs contains six steps (fig. S2): (i) draw the 2D pattern of the fabric sheets with AutoCAD 2020 (AutoDesk, USA); (ii) use the laser cutter to obtain the fabric sheets; (iii) adhere the air connector on the upper sheet; (iv) heat-seal the air chamber; (v) fabricate the X-crossing mechanism (including open the filaments, cross the filaments, and close the filaments with a connecting piece, i.e., ③ to ⑤ in Fig. 1B); and (vi) test the airtightness of the X-PAM. Typically, the material cost of the type 2 X-PAM is about $0.23 and can be fabricated within about 6 min (movie S2).

### Pressure sources and measurement system

The pressure source runs based on an industrial air compressor (ED-0204, 370 W, maximum of 0.8 MPa; Eidolon, China), a customized air source (Deli Group, China), two stainless air reservoirs (10 liters; Comeon, China), solenoid valves (N2V025-08 series; Chint Electric, China), and pressure sensors (MPX Series; Freescale Semiconductor, USA). The devices for static and dynamic measurement include a universal testing machine (68SC-2; Instron, USA), a laser displacement sensor (HG-C1400-P; Panasonic, Japan), and a flow sensor (MF-4003; Langfan, China). All these devices are controlled by the programmable real-time controller (microLabBox 1202; dSPACE, Germany) running with a frequency of 1 kHz. The corresponding supplementary information details more materials and apparatuses for these specific applications (Fig. 3A, figs. S1, S2, S5, S7, and S8, and Supplementary Texts).

### Modeling and simulation of X-PAMs

A fillet box is applied to approximate the pressurized chamber of the X-PAM and model the output force based on the principle of virtual work. This model presents the overall amplitude and variation trend of the output force, explains the actuation mechanism of X-PAMs, and can guide the design (fig. S3 and Supplementary Texts).

For the simulation of X-PAMs (using Python code–driven Abaqus/CAE Release 6.14-4), we process the fabric sheets as homogeneous continuum shells made of anisotropic lamina material and use a pair of spring components to substitute the filaments. Explicit dynamic steps are adopted to achieve quasi-static analysis, where the kinetic energy is maintained at no more than 5% of the system’s internal energy. This simulation method avoids some complex contacts and is a comprehensive paradigm of accuracy, feasibility, and efficiency (fig. S4 and Supplementary Texts).

## Supplementary Materials

### This PDF file includes:

Supplementary Text Figs. S1 to S9 Tables S1 to S6 Legends for movies S1 to S9 Legend for data S1 References
