# Supplementary material for: X-crossing pneumatic artificial muscles
Source: Sci Adv. 2023 Sep 20;9(38):eadi7133. doi: 10.1126/sciadv.adi7133 (PMC10511197; doi:10.1126/sciadv.adi7133)
Supplement: Supplementary file 1 — Supplementary Text Figs. S1 to S9 Tables S1 to S6 Legends for movies S1 to S9 Legend for data S1 References [file sciadv.adi7133_sm.pdf]

Science Advances
AAAS

Supplementary Materials for

X-crossing pneumatic artificial muscles

Miao Feng *et al.*

Corresponding author: Guoying Gu, guguoying@sjtu.edu.cn

Sci. Adv. **9**, eadi7133 (2023)
DOI: 10.1126/sciadv.adi7133

The PDF file includes:

Supplementary Text
Figs. S1 to S9
Tables S1 to S6
Legends for movies S1 to S9
Legend for data S1
References

Other Supplementary Material for this manuscript includes the following:

Movies S1 to S9
Data S1

Table S1. Properties of pneumatic artificial muscles and comparable objects in daily living.

PAMs	stimuli	max contraction ratio/%	max strain rate/%·s^{-1}	max actuation stress/MPa	max specific power/kW·kg^{-1}	work density /kJ·m^{-3}	efficiency/%	force-to-weight ratio/kN·kg^{-1}	specific force-to-weight ratio/N·kg^{-1}·kPa^{-1}	references
**X-PAMs**	**positive pressure**	**92.9**	**1603.0**	**10.8**	**5.7 (actuator mass 4.3 g, 100 kPa)**	**842.9**	**68.3**	**31.2 (150kPa@62.6%)**	**207.9 (150kPa@62.6%)**	**This work**
Muscle	ATP	40	500	0.8	0.323	40	40	-	-	(*1–6*)
Motor	electric	-	-	-	0.3	-	-	-	-	(*4*)
Jet engine	aviation kerosene	-	-	-	10	-	-	-	-	(*5*)
McKibben	positive pressure	36.4	800	3.4	10* (actuator mass 28 g, 600 kPa)	200	49	~11	~18.3	(*4, 11, 34, 38, 50–52*)
McKibben Variants	positive pressure	65	492*	33.7*	0.07*	42.2*	close to McKibben	~59.0 (300kPa@0%)	~196.8 (300kPa@0%)	(*38, 41, 53, 54*)
V-PAMs	vacuum	99.7	775* (no load)	15.3*	2.1	291.5*	22.7	2.5*	~43.4	(*26, 36, 55*)
Cavatappi	positive pressure	50	~66.7	0.7	1.42	0.38	45	4.9*	3.0*	(*23*)
Modular Multi-Chamber	positive pressure	71	~7.0	~5.8	-	-	22.9	-	-	(*43*)

The performance indicators for each kind of artificial muscle are not achieved by one design/actuator/configuration but are the best/representative values summarized from all actuators of the corresponding category.

X-PAMs (X-crossing Pneumatic Artificial Muscles), McKibben (McKibben Artificial Muscles), McKibben Variants (Variants of McKibben Artificial Muscles), V-PAMs (Vacuum-based Pneumatic Artificial Muscles), Cavatappi (Cavatappi Artificial Muscles), Modular Multi-Chamber (Pneumatic Artificial Muscles Based on Modular Multi-Chamber Soft Actuator).

-These data are not found or appliable.

*These data are not directly given by the corresponding references. We use the original data or read original data from graphs to calculate the properties.

~One data is lacking to calculate the property. We estimate the missing data according to the reference and calculate the property.

~/*These data can metric the performance of these actuators, but it is not reliable to directly cite these data as accurate values.

Table S2. Properties of various and typical artificial muscles.

Muscles	stimuli	max contraction ratio/%	max strain rate/%·s^{-1}	max actuation stress/MPa	max specific power/kW·kg^{-1}	work density /kJ·m^{-3}	efficiency/%	references
**X-PAMs**	**positive pressure**	**92.9**	**1603.0**	**10.8**	**5.7**	**842.9**	**68.3**	**This work**
HASEL	voltage	24	7400	0.3	0.6	64.4	21	(*3, 4, 46, 56*)
SCP/TSA	chemical/thermal/humidity/ions	80	80	84	27.9	3025	5.4	(*10, 11, 22, 31, 57, 58*)
DEA	voltage	99.8	34000	7.7	5	3400	90	(*2, 11–14, 59*)
HGA	light/chemical/thermal	45	2	0.29	0.0002	460	0.2	(*2, 7, 60*)
SMA	thermal	50.7	300	700	50	10000	16	(*2, 4, 11, 61*)
CNT	voltage/thermal	33	120	115	0.27	1000	55	(*4, 11, 62*)
Piezo	voltage	10	-	300	0.17	2550	90	(*6, 11, 12*)
LCE	voltage/light/thermal	40	90	0.45	0.29	150	75	(*2, 16, 63–65*)
CP	voltage	12	12	34	0.15	100	18	(*2, 4, 11*)
FP	voltage	7	2000	45	4	1000	80	(*2, 4*)
IPMC	voltage	40	9	30	0.24	5.5	2.9	(*2, 4, 11*)

The performance indicators for each kind of artificial muscle are not achieved by one design/actuator/configuration but are the best/representative values summarized from all actuators of the corresponding category.

X-PAMs (X-crossing Pneumatic Artificial Muscles), HASEL (Hydraulically Amplified Self-healing Electrostatic Actuators), SCP/TSA(Supercoiled Polymer/Twisted String Actuators), DEA (Dielectric Elastomer Actuators), HGA (Hydrogel Actuators), SMA (Shape Memory Alloys), CNT (Carbon Nanotube Actuators), Piezo (Piezo-actuators), LCE (Liquid Crystal Elastomers), CP (Conductive Polymers), FP (Ferroelectric Polymers), IPMC (Ionic Polymer-metal Composites).

-These data are not found or applicable.

Supplementary Texts

·Properties of materials

TPU-coated N210D Nylon woven fabric fabricates X-PAMs (**Fig. S1A**). We present the properties of this in-plane anisotropic material in **Fig. S1B** and **Table S3**, which are obtained from 7 specimens (200 mm × 40 mm) with the method adopted from reference (*66*). In the experiments, each specimen is clamped and stretched by the universal testing machine from 0% to 15% with a speed of 30 mm·min^{-1} and a pre-tension force of 0.5 N. Separate specimens are tested for the breaking strength and the elongation at break. As a metric of the capability to withstand pressure, the seam strength of the heat-sealed fabric is also obtained with the same method. It is worth noting that, as a component of composite material, TPU in our fabric is used to provide a thermoplastic connection and improve the strength of the matrix.

Table S3. Properties of TPU-coated N210D Nylon woven fabric

Materials: TPU-coated N210D Nylon woven fabric			
yarn density/Tex	116		
grammage/g · m^{-2}	250-300, aver. 275		
thickness/mm	0.2 (with 0.1 mm TPU included)		
density/kg · m^{-3}	1250-1500, aver. 1375		
u_{12}	0.412	G_{12}/MPa	14.8
u_{21}	0.326	E_{45}/MPa	54.1
E_1/MPa	343.1	E_2/MPa	134.6
σ_{b1}/MPa	70.6	σ_{b2}/MPa	43.2
δ_{b1}	0.28	δ_{b2}	0.23
seam strength-0°/ N· m^{-1}	13155.4	seam strength-90°/ N· m^{-1}	10014.6

u:Poisson ratio; E: Elastic Modulus; G: Shear modulus; σ_b: Breaking strength; δ_b: Elongation at the break.

·Geometric parameters of X-PAMs

The geometry of X-PAMs is shown in **Fig. 2A** and **B** and **Fig. S2A**. These geometric parameters include L (the length of the chamber when folded), W (the width of the air chamber), N (the number of filaments), w_{seam} (the polytechnical width of the seam), w (the width of each filament).

The outermost filaments belonging to the wider side ($N + 1$ filaments) have a width of $2w$, which makes the actuation of X-PAMs more stable.

The width of the filament meets with

$$(2N + 3)w = W + 2w_{\mathrm{seam}} \tag{S1}$$

The area of the air chamber A

$$2WL = A \tag{S2}$$

The geometric parameters of exemplary X-PAMs in this work are presented in **Table S4**, where the length of adjustable tendons is uniformly set for clamping convenience in these experiments.

Table S4. Geometric parameters of X-PAMs

X-PAMs	type 1	type 2	type 3
A/mm^2		5000	
$L{:}W$	2:1	1:1	1:2
L/mm	70.17	50.00	35.36
W/mm	35.36	50.00	70.71
$w_{\mathrm{seam}}/\mathrm{mm}$		4.61	
N		4	
length of adjustable tendons		$L/2 + 20$	

·Static characterization of X-PAMs

For each type of X-PAM, there are 5 specimens tested in their static characterization with the following steps.

1) Clamp the specimen (no stretching) using the universal test machine and clear the force.

2) Adjust the slide until a pre-tension force of 0.5 N is applied to the specimen.

3) Clear the displacement and the force.

4) Apply a constant pressure series of 0 : 15: 150 kPa on the specimen, where each constant pressure is kept for 2.5 s.

5) If the forces for all pressure are less than 0.5 N, go to step-6. If not, move the upper clamp downward for 5 mm and repeat step-4.

6) One time of measurement is completed. Reset the slide to the original point and repeat step-1 for a new measurement. Each specimen has 3 times of measurements.

The force data of the latter 1.5 s for each constant pressure are extracted and smoothed to calculate the average output force. Multiple specimens and multiple measurements reduce random errors from manufacturing and testing.

·Modeling of X-PAMs

·Caculations of geometric parameters

We approximate the unfolded pressurized chamber of X-PAM as a fillet box, as shown in **Fig. S3A**. The folding of X-PAM results in deformation, including creases, mainly around the central parts. Therefore, the radius r of the fillet box is assumed unchanged when bending under specific pressure.

The parameters of the fillet box before pressurization are W_0-chamber width, L_0-chamber length, and t-thickness. Accordingly, The parameters of the fillet box after pressurization are W_b-chamber width, L_b-chamber length, and r-fillet radius.

We first assume that the fabric sheets are inextensible and that these parameters meet with

$$\begin{cases} W_\mathrm{b} + \pi r = W_0 \\ L_\mathrm{b} + \pi r = L_0 \end{cases}, r \in \left[0, \min\left(\frac{W_0}{\pi}, \frac{L_0}{\pi}\right)\right] \tag{S3}$$

The inside volume of the air chamber is

$$V(r) = 2W_\mathrm{b}L_\mathrm{b}r + \pi r^2(W_\mathrm{b}+L_\mathrm{b}) + \frac{4}{3}\pi r^3 \tag{S4}$$

The chamber inflation will introduce a work of $-\int pdV$. To let the system stay at a minimum energy state, the fillet radius r should be the value that makes the largest volume $V_{\max}(r)$, which can be obtained.

·Correcrion of elasticity

Actually, the elasticity of fabric sheets cannot be entirely ignored. We correct the geometric parameters using the r obtained from the preliminary calculations. Similar to the situation in the classical thin-walled cylinder, there exists

$$\begin{cases} p(\pi r^2+2W_\mathrm{b}r) = \sigma_\mathrm{L}(2W_\mathrm{b}+2\pi r)t \\ \sigma_\mathrm{L} = \varepsilon_\mathrm{L}E_1 \\ p(\pi r^2+2L_\mathrm{b}r) = \sigma_\mathrm{W}(2L_\mathrm{b}+2\pi r)t \\ \sigma_\mathrm{W} = \varepsilon_\mathrm{W}E_2 \end{cases} \tag{S5}$$

Where E_1, E_2, p, σ_{L}, σ_{W}, ε_{L}, and ε_{W} are the elastic modulus in direction 1, the elastic modulus in direction 2, pressure, stress in direction 1, stress in direction 2, strain in direction 1, and strain in direction 2.

The corrected W and L are

$$\begin{cases} W_0{}' = (1+\varepsilon_{\mathrm{W}})W_0 \\ L_0{}' = (1+\varepsilon_{\mathrm{L}})L_0 \end{cases} \tag{S6}$$

With the corrected $W_0{}'$ and $L_0{}'$, we can obtain the corrected r' by substituting them into Eq. (S3)-(S4). For simplification, we still denote these parameters after correction as W_0, L_0, and r in the following paragraph.

·Volume change

A chamber folding about its central axis has a volume change from its unfolding state (**Fig. S3B** and **C**). The volume change at the upper sheet side includes the disappearing and increasing parts, which are comparatively small and ignored here.

We assume that the volume change is $\Delta V - \mathrm{OCD}$ composed of $\Delta V_1 - \mathrm{OBD}$ and $\Delta V_2 - \mathrm{OBC}$. The arc $\widehat{\mathrm{AC}}$ with radius R tangents to the crease $\overline{\mathrm{OB}}$ and the chamber surface $\overline{\mathrm{BC}}$. For a bending angle θ from the unfolding state, it has

$$R tan\frac{\pi-\theta}{4} + r tan\frac{\theta}{2} = \frac{\pi-\theta}{2}R \tag{S7}$$

The crease radius is

$$R = \frac{r tan\frac{\theta}{2}}{\frac{\pi-\theta}{2} - tan\frac{\pi-\theta}{4}}, \theta \in [0, \theta_{\mathrm{cr}}] \tag{S8}$$

When the tangent point C grows to touch the fillet arc, the chamber enters the flatting state from the folding state. Since the length of the sheet keep unchanged before and after folding, the critical angle θ_{cr} meets with

$$\frac{\pi-\theta_{\mathrm{cr}}}{2}R = \frac{L_{\mathrm{b}}}{2} \tag{S9}$$

We denote the arc projection length on the chamber section $\overline{\mathrm{CH}}$ as x_{m}

$$x_{\mathrm{m}} = R\left(1 - sin\frac{\theta}{2}\right) \tag{S10}$$

The total volume change should be

$$\Delta V = -2(\Delta V_1 + \Delta V_2) \tag{S11}$$

Where

$$\begin{cases} \Delta V_1 = \int_0^{\frac{\pi}{2}} (W_\mathrm{b} + 2rcos\alpha)\left(rsin\alpha tan\frac{\theta}{2}\right) d(rsin\alpha) = 2\left(\frac{1}{2}W_\mathrm{b} + \frac{2}{3}r\right) r^2 tan\frac{\theta}{2} \\ \Delta V_2 = \int_0^{x_\mathrm{m}} (W_\mathrm{b} + 2\sqrt{r^2 - (r-x)^2})\left(rtan\frac{\pi - \theta}{4} + xtan\frac{\theta}{2} - \sqrt{R^2 - (R-x)^2}\right) dx \end{cases} \tag{S12}$$

The chamber volume can be denoted as

$$V = \Delta V + V_0, (V_0, \text{the initial volume}) \tag{S13}$$

·Output force

We first calculate the resistant torque M produced by the chamber when it is folded, and then, the linear output force F is further obtained.

According to the principle of virtual work (**Fig. S3D**), it has

$$Md\theta + pdV = 0 \tag{S14}$$

$$M = -p\frac{\partial V}{\partial \theta} = -p\frac{\partial \Delta V}{\partial \theta}, \theta \in [0, \theta_\mathrm{cr}] \tag{S15}$$

For the flatting state, we use a linear estimate for calculating the torque (*67*) as

$$M = \left.\frac{dM}{d\theta}\right|_{\theta=\theta_\mathrm{cr}} (\theta - \theta_\mathrm{cr}) + M_\mathrm{cr}, \theta \in [\theta_\mathrm{cr}, \pi] \tag{S16}$$

We have the output force

$$pdV - Fdx = 0 \tag{S17}$$

$$F = \frac{pdV}{dx} = \frac{p\,{}^{dV}/_{d\theta}}{{}^{dx}/_{d\theta}} = -\frac{M}{{}^{dx}/_{d\theta}} \tag{S18}$$

As shown in **Fig. S3E**, for the relationship between x and θ, there is some difference between considering chamber expansion (the lower half diagram) and not considering that (the upper half diagram). Since the chamber is compressible, the actual deformation should be some compromise between these deformations. Here, we simply take an average to estimate the relationship.

$$x = \frac{1}{2} \cdot 2\left[\frac{L_\mathrm{b}}{2}cos\frac{\theta}{2} + r\left(\frac{\pi}{2} - \frac{\theta}{2}\right)\right] + \frac{1}{2} \cdot Lcos\frac{\theta}{2} \tag{S19}$$
$$= \frac{1}{2}L_\mathrm{b}cos\frac{\theta}{2} + \frac{1}{2}r(\pi - \theta) + \frac{1}{2}Lcos\frac{\theta}{2}$$

With the decrease of θ (chamber expanding, actuator contracting), the torque M will decrease, which may result in a smaller output force. However, on the other hand, the decrease of θ can improve the output force. As a result, the output force can be maintained at a high level for a wide range of contraction ratios, even when the contraction ratio is large.

When $\theta = 0$, the output force theoretically reaches the maximum value and then will instantly become 0 when continuing contracting. However, the actual chamber has some elasticity, and the output force should be continuous. Therefore, we introduce a linear force estimate between the maximum force and the theoretical maximum contraction ratio (**Fig. S3F** to **H**).

·Simulation of X-PAMs

We regard the fabric sheets as homogeneous continuum shells and use Python code-driven Abaqus/CAE (Release 6.14-4) to simulate X-PAMs (**Fig. S4**). Actual simulation shows that a realistic model with all details of design involved consumes lots of time and usually fails since there are geometric nonlinearity, complicated contact, and shell instability. Therefore, we adopt a pair of linear springs to serve as the filaments of an X-PAM, whose spring coefficients are easy to obtain by the law of serial and parallel connections of springs. Additionally, a rigid plate acts as the unilateral constraint from the filaments for the chamber of an X-PAM (the belly part) (**Fig. S4A**).

To avoid non-convergence, explicit dynamic steps with slow loading are incorporated to ensure a quasi-static analysis where the kinetic energy is no more than 5% of the internal energy for the system (*68*) (**Fig. S4B**). Specific details of the configuration are as follows.

·Materials and Section

Material: lamina, $E_1 = 343.1$ MPa, $E_2 = 134.6$ MPa, $\nu = 0.37$, $G_{12} = G_{13} = G_{23} = 14.8$ MPa (*69*). These primary parameters of the fabric sheet are referred to in **Table S3**.

Section: continuum shell, homogeneous; shell thickness 0.2 mm; Simpson integration (15 points).

·Parts and Assembly

All the geometry and assembly relationships are the same as the actual situation where the X-PAM is clamped. A tie constraint connects the seams of the upper sheet and the lower sheet (specify distance 1 mm).

·Interactions

Properties: tangential frictionless; normal "hard " contact.

Interactions: self-contact of the lower sheet; mutual contact between the lower sheet belly and the rigid plane; mutual contact between the lower sheet belly and its seam.

·Steps

BCs: The pin-1 of the spring have $u_{\mathrm{x}}, ur_{\mathrm{z}}$ FREE and otherwise SET. The pin-2 is fixed to the rigid plane. All degrees of freedom for the rigid plane are fixed but u_{y} (**Fig. S4A**).

Loads: the pressure applied is shown in **Fig. S4A**.

Steps: dynamic, explicit; Nlgeom ON; automatic incrementation; Improved Dt method ON; mass scaling with a target time increment of 2×10^{-5} s to accelerate the simulation.

·Mesh

Element shape: QUAD; algorithm: MEDIAL_AXIS.

All the unmentioned parameters are default; please see details in the attached exemplary Python code and "cae" project file (Note whether these codes or files can directly run depends on the software version or configuration). The simulated output force in real-time has some fluctuation in **Fig. S4C**, and we extract the force of the latter 2 s for each pressure to calculate the average output force.

·Generation and control of pressure

·Air supply system

The air supply system includes an industrial air compressor (ED-0204, 370 W, max. 0.8 MPa, Eidolon, China), a customized air source with a group of regulators embedded (Deli Group, China), a controller (microLabBox 1202, dSPACE, Germany), and a computer (**Fig. S5A**). The air compressor is connected to the customized air source and intermittently works to maintain the air supply. Further, the customized air source, controlled by the microLabBox, regulates the input pressure from the compressor into the desired output

pressure. In all our experiments for data, the average total power of the measuring system is about 100 W (except the PC), according to our experience (since the operation is intermittent, and the power consumption of some internal devices is not entirely known).

We should mention that, for potential portable deployment instead of our experiments in the laboratory, micro pumps can be adopted to reduce system volume, noise and power consumption. As shown in **Fig. S5B**, the micro pump (12 V, fspump, China) with small airflow consumes 0.7-1.4 W (reaching the max contraction in 6.0 s), and the micro pump (12V, KZP-PE, Kamoer Fluid Tech Co., Ltd., China) with a comparatively large airflow consumes 7.9-10.4 W (reaching the max contraction in 1.8 s). These examples are for reference, and more different pumps/air reservoirs are optional according to specific requirements.

·Generation of constant pressure

A constant pressure is required for static characterization of X-PAMs to obtain the force-displacement relations. The generation of constant pressure should be accurate, and there is no special requirement for its generation speed. We directly connect the air supply system (functional to produce desired pressure but with some unestimated error) to the actuator to generate the specified pressure. A simple PID controller is manually tuned to reduce the error (the white block in **Fig. S9A**), where p_{d} is the desired pressure, and p is the actual pressure (feedback) in real-time.

·Generation of sinusoidal pressure

A sinusoidal pressure is required for the durability test of X-PAMs and the walking of the jumping robot, which shares the same apparatus with the generation of constant pressure as described above. We can easily generate a sinusoidal pressure by inputting the expected pressure curve.

·Generation of step pressure

Step pressure is required by the step response of X-PAMs, the jumping robot, and the applications presented. It is impossible to produce an ideal step stimulus using physical systems, and particularly challenging for pneumatic systems to produce step pressure. However, creating an approximate step pressure is worthwhile since step pressure as a kind of normative stimulation can provide comparability.

Here, we adopt a pair of stainless air reservoirs (10 L, max. 0.7 MPa) to generate step pressure (*28*), as shown in **Fig. S5D**. These two air reservoirs are connected to the customized air source, of which the high-pressure reservoir is kept at 400 kPa for high-speed pressure generation, and the low-pressure reservoir is kept at the desired step pressure of 100 kPa here for maintaining the pressure in the actuator.

We can generate the step pressure through the timings of solenoid valves with the following steps (**Fig. S5E** and **F**).

1)Pressurize the air reservoirs and maintain their pressures at expected values.

2)Turn on the valve of the high-pressure reservoir (valve 1).

3)When the pressure in the actuator reaches the expected step pressure, simultaneously turn off valve 1 and turn on the valve of the low-pressure reservoir (valve 2).

4)After a specified period (determined by specific application requirements), turn off valve 2 and turn on valve 3 to vent the compressed air out to the ambient environment.

Inevitably, there is some overshooting and oscillation, but the pressure can quickly back to the expected pressure due to the connection with the low-pressure reservoir (**Fig. 3B** and **Fig.S5F**). This method can reach the desired step pressure using no more than 0.2 s in all the experiments for different X-PAMs or applications.

·Calculations of mechanical performance

We denote the following real-time data from sensors at time t: p-absolute pressure in the air chamber, F-output force, x-displacement to the initial point, Q-mass flow of compressed air into the X-PAM. And we have parameters: m-mass of the X-PAM, m_{load}-total mass of the load, A_{m}-section area of materials (the filaments), V_{m}-material volume of the X-PAM, V- volume of the air chamber.

·actuation stress

$$\frac{F}{A_{\mathrm{m}}} \tag{S20}$$

·force-to-weight ratio

$$\frac{F}{m} \tag{S21}$$

·specific force-to-weight ratio

$$\frac{F}{p_{\mathrm{gauge}} m} \tag{S22}$$

Where p_{gauge} is the gauge pressure of p.

·strain

$$\frac{x}{2(L+w_{\text{seam}})} \tag{S23}$$

·stain rate

$$\frac{\dot{x}}{2(L+w_{\text{seam}})} \tag{S24}$$

·power

$$P = F\dot{x} \tag{S25}$$

·power density

$$P_{\text{m}} = \frac{F\dot{x}}{m} \tag{S26}$$

·work density

$$W_{\text{m}} = \frac{1}{V_{\text{m}}}\int_{0}^{x} F\,dx \tag{S27}$$

·efficiency

$$\eta = \frac{W}{E} = \frac{\int_0^x F dx}{\int_{p_0}^{p} p_{\text{gauge}} dV} \times 100\% \tag{S28}$$

The chamber volume can be obtained by

$$pV = \xi \int_0^t Q dt \Rightarrow V = \frac{\xi}{p}\int_0^t Q dt \tag{S29}$$

Where the constant ξ (*28*) is

$$\xi = \frac{\rho RT}{M} \tag{S30}$$

The parameters ρ, R, T, M are the air density under the standard conditions (1.293 g·L^{-1}, 0 °C, 101.325 kPa), the ideal gas constant (8.314 J·mol^{-1}·K^{-1}), the absolute temperature, and the molar mass of air (28.9634 g·mol^{-1}), respectively. Regarding a specific measurement, we use a data clip to calculate these indicators (clip start point: the beginning of airflow into the actuator; clip end point: the first time the actuator reaches its final contraction ratio).

·Features of the deflating process

In the experiments characterizing the dynamic properties of X-PAMs, we apply a step pressure of 100 kPa to the actuator and maintain it for 5 s. Then, the solenoid valve for venting is opened, and the compressed air flows into the atmosphere (**Movie S3**, **Fig. S5D**

to **F**). Harnessing the same calculation method mentioned above in "Calculations of mechanical performance," we obtain the features of the deflating process for the type 2 X-PAM (**Fig. S6C** to **E**). Regarding a specific measurement, a data clip is adopted for calculation, which starts from the deflating to the moment when the pressure inside the air chamber is less than 2 kPa.

The experimental results show that the strain rate, specific power, and work density are -89.2 $\%\cdot s^{-1}$ (average), -598.1 $\%\cdot s^{-1}$ (peak), -0.3 $kW\cdot kg^{-1}$ (average), -2.9 $kW\cdot kg^{-1}$ (peak), and -329.9 $kJ\cdot m^{-3}$ (**Fig. S6D** and **E**). These negative signs "-" mean that force and displacement are in opposite directions during deflation.

We should mention that, unlike the step pressure during inflating, the pressure curve during deflating is not standard/normative and depends on various factors such as the actuator, load, air passage, and valve. Therefore, these results are a good reference for metering the deflating features but can only provide limited comparability.

·Robotic elbow, jumping robot, and soft gripper

·Robotic elbow

The robotic elbow, fixed on a cylindrical 3D-printing base (top height 80 cm, resin, Yungong Industrial Technology Co., Ltd, China) and a tripod, is fabricated by patterned acrylic plates (thickness 6 mm, Deyao Plastic Material Factory, China), where its upper arm and forearm length is 210 mm and 275 mm, respectively (**Fig. S7A** to **C**). The anchor point of artificial muscle (the type 3 X-PAM is used here) is located on the forearm and 40 mm from the elbow joint. With this robotic arm, the tests for actuation speed and capability of load withstanding are completed in an indoor laboratory while the experiments to vertically throw a ping-pong ball (initial height 30 cm, 2.5 g, diameter 40 mm, DHS, China) are implemented in an open space, an lawn. Here, an Arduino (ATMEGA328, Arduino, Italy) replaces the controller dSPACE for portability for outdoor experiments.

Five specimens are adopted in the throwing experiments of ping-pong, and three trials are repeated for each specimen. We then obtain the thrown height of the ping-pong ball through image-based measurement (**Fig. 4A**). And indirectly, we use the final height to inversely estimate the initial velocity of the ping-pong with the following method.

The dragging force is

$$F = C_{\mathrm{d}} \cdot \frac{1}{2}\rho V^2 A_{\mathrm{ball}} \tag{S31}$$

The kinetic equation is

$$F + mg = ma \tag{S32}$$

The drag coefficient C_d for a smooth sphere is referred to empirical formula (*70*)

$$C_\mathrm{d} = \frac{24}{Re} + \frac{2.6\left(\frac{Re}{5.0}\right)}{1 + \left(\frac{Re}{5.0}\right)^{1.52}} + \frac{0.411\left(\frac{Re}{2.63 \times 10^5}\right)^{-7.94}}{1 + \left(\frac{Re}{2.63 \times 10^5}\right)^{-8.00}} + \frac{0.25\left(\frac{Re}{10^6}\right)}{1 + \left(\frac{Re}{10^6}\right)} \tag{S33}$$

Where Reynolds number Re is

$$Re = \frac{\rho V D}{\mu} \tag{S34}$$

The variables and parameters above $\rho, V, A_\mathrm{ball}, m, g, a, \mu, D$ are air density (1.204 kg·m^{-3}, 20°C), the velocity of ping-pong, the cross-sectional area of ping-pong, the mass of ping-pong (2.8 g), gravitational acceleration constant (9.83 m·s^{-2}), acceleration, the dynamic viscosity of air (18.13×10^{-6} Pa·s, 20 °C), and the diameter of ping-pong (40 mm).

·Jumping Robot

The jumping robot (337.3 g) fabricated by acrylic plates (thickness 5 mm, Deyao Plastic Material Factory, China) is generated by the parallel mechanism with a diamond shape (**Fig. 4B and C**, and **Fig. S7D**), whose joints are connected by steel screws. When the horizon diagonal of the "diamond" mechanism gets shortened due to the contraction of a central actuator (the type 2 X-PAM is used here), the jumping robot stands up from its resting state (49 mm). If the contraction strain rate is high enough, the jumping robot will get enough impulse from the ground reaction force to jump up. In particular, the foot of the jumping robot is carved into unidirectional jagged in case of slipping, which grants its ability to walk.

The jumping robot is targeted to horizontally walk 60 cm and then vertically jump up to eat (touch) the plastic strawberry model (7.2 g, diameter 30 mm, length 65 mm, hang up to a height of 80 cm) using its "mouth"/gripper with double-sided tape. For the walking task, we apply a sinusoidal pressure of 0.1 Hz from 0 to 50 kPa to generate the steps. When the jumping robot reaches just below the strawberry model, a step pressure of 100 kPa is stimulated.

·Soft Gripper

The soft gripper (1.9 g) aims to verify the scalability of X-PAMs (**Fig. 4D** and **Fig. S7E**). Three small-scale X-PAMs with a width of 15 mm are cascaded into a circle, and one

side of them is heat-sealed together, forming a funnel to envelop and grasp objects when these X-PAMs contract to a smaller radius. Another small-scale X-PAM works as a serially-connected contraction unit to lift objects. All the X-PAMs are coupled and actuated by one air tube with a pressure of 100 kPa.

We manually put the object inside the loose gripper for each grasping task and then pressurize it. The information on these objects is presented in **Table S5**.

Table S5. Objects for grasping task of soft gripper

Objects	Mass/g	Dimensions/mm
cherry skewer	15.4	-
ping-pong ball	2.5	$D: 40$
hedgehog ball	28.9	$D: 60$
egg	56.9	$D \times H: 43.5 \times 53.5$
cup	85.4	$D \times H: 60 \times 145$
lemon	11.7	$D \times H: 50 \times 75$
purple sweet potato	6.7	$D \times H: 55 \times 65$
sealing tape	10.8	$D \times H: 55 \times 16$
strawberry model	7.2	$D \times H: 30 \times 65$
measuring tape	86.3	$D \times H: 60 \times 28$
stapler	35.8	$L \times W \times H: 65 \times 25 \times 45$
microcontroller	27.8	$L \times W \times H: 75 \times 50 \times 12$
soft component	64.7	$L \times W \times H: 75 \times 40 \times 40$

D: diameter, L: length, W: width, H: height

·McKibben artificial muscles for comparisons

McKibben artificial muscles are fabricated mainly by an air chamber inside and an outside sheath (**Fig. S7A** to **C**). Here, to avoid pressurization resistance from the air chamber, we must choose an air chamber that is large enough and can easily and loosely inflate to fill the sheath. To ensure that the McKibben muscles are comparable with X-PAMs, we try to keep two primary geometry parameters at the resting state the same/or close as possible: the initial active length of the actuator and the section circumference of the actuator.

For the counterpart of the type 2 X-PAM (length 150.64 mm, circumference 100.00 mm), the basic McKibben muscle (balloon+nylon sheath) has a length of 150.64 mm and a circumference of 94.25 mm, which is used in the jumping robot for comparison.

For the counterpart of the type 3 X-PAM (length 79.93 mm, circumference 141.44 mm), the basic McKibben muscle (balloon+nylon sheath) has a length of 79.93 mm and a circumference of 141.30 mm, which is used in the robotic elbow for comparison.

More comparative McKibben muscles with different materials are presented in **Fig. S7B**. These materials are balloons, plastic chambers, nylon sheaths and fabric sheaths. Their geometric parameters are listed in the following **Table S6**.

Table S6. Component parameters of different materials

Materials	Circumference/mm	initial length/mm
**X-PAM (type 3, baseline)**	**141.44**	**79.93**
balloon	inflate to the max. 18 inches **(make sure large enough to easily and loosely inflate to fill the sheath)**	(elastic&spherical)
plastic chamber	180.00 **(make sure large enough to easily and loosely inflate to fill the sheath)**	79.93
nylon sheath	141.30	79.93
fabric sheath	140.00	79.93

It is important to note that these circumferences cannot be maintained entirely the same since the available parameters of commercial materials are not arbitrary.

·Configuration of extremes environments and durability test

·Configuration of extremes environments

We use one X-PAM (type 2) to investigate its performance in various environments (**Fig. 5**), whose detailed configurations are as follows.

Cold environment: The refrigerator (BCD-308WPZME(E), Midea Group, China) is kept at no more than -20°C. The rubber duck (57.8 g) and X-PAM are placed inside for no less than 5 min, and then we manually pressurize the actuator to 100 kPa with the device in **Fig. S8B**.

High-temperature environment: The thermostat (DZ-2BCIV, Taist Instrument Co., Ltd, China) is kept at no less than 100°C. The rubber duck and X-PAM are placed inside for no less than 5 min, and then we manually pressurize the actuator to 100 kPa.

Underwater pneumatic actuation: The X-PAM with a load of 1 kg is hung and immersed into a water tank (600 mm × 400 mm × 450 mm, water depth of about 300 mm). Then we manually pressurize the actuator to 100 kPa.

Underwater hydraulic actuation: The X-PAM is immersed into the water tank with one adjustable tendon held in place by a weight of 5 kg and the other dragging a weight of 0.5 kg. We use a micro water pump (12VDC, Baolian, China) to drive the system. The hydraulic output force is measured with the device in **Fig. S8A** and **C**.

Vehicle rolling test: After cleaning and drying, the deflated X-PAM is placed ahead of the vehicle wheels (1500 kg). One researcher drives the car at about 5-10 $km \cdot h^{-1}$, and the front and rear wheels slowly roll over the X-PAM in succession. Just after the rolling is over, another researcher manually pressurizes the actuator to 100 kPa. To further investigate the performance of the X-PAM rolled at the inflated state, the researcher maintains the X-PAM at no less than 100 kPa and rolls over it (**Movie S8**, *Note: this is a supplementary experiment after a long time. The car is changed with a mass of about 2500 kg since we cannot find the same one).

·Configuration of durability test

We use one X-PAM (type 2) to investigate its long-term performance (**Fig. 5G** and **H**).

For the force durability test, the X-PAM is fixed at a contraction ratio of 50%. A sinusoidal pressure of 0.5 Hz from 0 to 100 kPa is applied until the cycles are more than 5000 times. We record the output force during the test.

For the displacement durability test, the X-PAM is clamped with a load of 2 kg. The same sinusoidal pressure of 0.5 Hz from 0 to 100 kPa is applied until the cycles are more than 5000 times. We record the displacement of the actuator during the test.

·Implementation and measurement for the variants of X-PAMs

The details for the variants of X-PAMs are as follows (**Fig. 6**), and please see the attachments for mechanical drawings.

·X-PAM couple

Two actuators of the type 2 X-PAM comprise an X-PAM couple by symmetrically connecting and sharing the same filaments (**Fig. 6A**, **Movie S9**). Its force-displacement relationship is obtained from 5 specimens with the same method used for a single X-PAM, but the steplength is set at 3 mm (shorter than that in the static characterization for better spatial resolution).

·Serial and parallel X-PAMs

Three actuators of the type 2 X-PAM are end-to-end cascaded into serial X-PAMs, and three serial X-PAMs comprise parallel X-PAMs (**Fig. 6B** and **C**).

·Small-scale and large-scale X-PAM

The material to fabricate the small-scale X-PAM (0.3 g, width 15 mm) is 0.1 mm TPU-coated N66 woven fabric for better softness. The number of filaments for small-scale X-PAM N is 1 for the convenience of manual fabrication. We should note that the width of the seam here w_{seam} is not 4.61 mm, but we try to manually make it as narrow as possible (about 1.5 mm). The structure of the large-scale X-PAM (49.3 g, width 210 mm) is the same as that of the original X-PAM. (**Fig. 6D** and **E**)

·Asymmetric X-PAM actuating the scissor mechanism

The chamber of the asymmetric X-PAM (4.9 g) is an isosceles trapezoid (upper base 30 mm, lower base 120 mm, height 60 mm) whose output torque at 40°, 60°, and 80° (**Fig. 6F**) is obtained by the torque measurement platform (**Fig. S8A**) with the same pressurization method in static characterization. Here, the measurement platform mainly comprises a precision rotation guide (RSP 125-L, Dongguan Shengling Precision Machinery, China) and a torque sensor (TTF 400, FUTEK Advanced Sensor Technology, Inc., USA).

·Ring-shaped X-PAM

The ring-shaped X-PAM (6.3 g, length 120 mm, diameter 25 mm) is fabricated by two rectangular fabric sheets, one inside the cylindrical surface and the other outside (**Fig. 6G**). To avoid these two sheets being stuck under heat, a piece of barbecue release paper is inserted between the seams on both surfaces (blue and yellow). Two strong fishing threads pass through the center, working as the filaments and adjustable tendons.

·Origami-combined X-PAM

The origami-combined X-PAM (17.9 g, **Fig. 6H**) comprises an original X-PAM and two PET (polyethylene terephthalate) sheets (thickness 0.3 mm, see the planar mechanical drawings in Data). The filaments of X-PAM are inserted through the carved seam (width 1.5 mm) in the middle of the PET sheet. The edge of the PET sheet near the chamber is fixed to the edge of the chamber on the same side through a set of pinholes and thread. And the other side is fixed to the adjustable tendons.

· "8"-shaped X-PAM

The "8"-shaped X-PAM (10.4 g, **Fig. 6I**) comprises an original X-PAM and an additional chamber, whose components are the same as an X-PAM couple. However, for the X-PAM couple, the edges of the two chambers are directly connected to form a "diamond" shape (**Fig. 6A**). In contrast, for the "8"-shaped X-PAM, the edges of the additional chamber are connected to the ends of the filaments (**Fig. 6 I**).

·Tracking of force and displacement

We test the tracking performance of the type 2 X-PAM in the force and displacement (**Fig. S9A** to **C**). The white block in **Fig. S9A** is a PID controller with pressure feedforward to control the pressure in the actuator chamber. Accordingly, the blue block is another PID controller with force feedforward to control the output force of X-PAM. All the PID parameters are manually tuned, and these feedforward functions are obtained by simply measuring a group of static input and output.

For force tracking, we clamp the X-PAM at the contraction ratio of 50% and give out a desired sinusoidal force of 0.5 Hz from 10 to 40 N.

For displacement tracking, the structure of the control diagram is the same as that adopted in force tracking. The only adjustment is substituting the input and the feedforward function with x_{d} and $\varphi_{\mathrm{x}}^{-1}(x_{\mathrm{d}})$. Here, we let the X-PAM with a load of 2 kg track a desired sinusoidal displacement of 0.5 Hz from 10 to 40 mm.

·Frequency response of force and displacement

The frequency response (type 2 X-PAM) of force and displacement is presented in **Fig. S9D** and **E**. In the experiments, we apply a sinusoidal pressure of 0-100 kPa to the actuator and sweep a frequency from 0.01 Hz to 5 Hz with a rate of 0.01 Hz/s. The force and the

displacement as the outputs are recorded. It is important to note that the amplitude of supplied pressure during sweeping frequency significantly decreases when the frequency exceeds approximately 1 Hz due to the inherent challenges associated with pneumatic sources operating at high frequencies.

For the frequency response of force, we clamp the X-PAM at the contraction ratio of 50%. The Bode diagram shows that the cut-off frequency is about 3.25 Hz (-3 dB), which is a satisfying result for PAMs (**Fig. S9D**).

For the frequency response of displacement, a load of 2 kg is applied to the X-PAM. The Bode diagram shows that the cut-off frequency is about 0.61 Hz (-3 dB), located within the overall performance of PAMs (**Fig. S9E**).

The working bandwidth of PAMs depends on complicated factors, including actuator structure, chamber volume (air consumption), pneumatic source, air passage, and load applied. Though our X-PAMs, with the structure of an X-crossing mechanism, demonstrate the capability to achieve a high actuation speed (strain rate of contraction, $1603.0\%\cdot s^{-1}$) in dynamic tasks such as robotic elbow and jumping robots, we should mention that it remains challenging to effectively harness this advantage for continuous dynamic responses. Potential methods include optimizing air consumption, improving the speed of air exhaust, or incorporating the air recirculation mechanism according to specific application requirements (*28*).

Supplementary figures

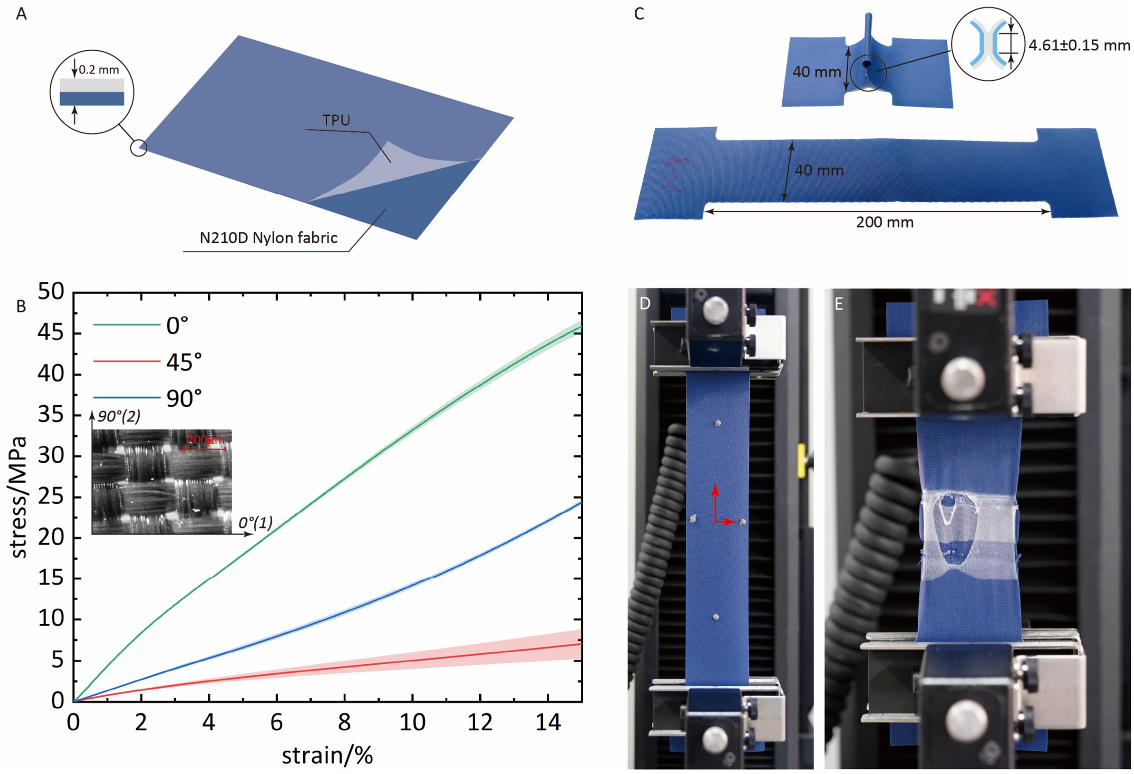

Fig. S1. Materials and testing configuration. (A) Structure of N210D nylon fabric with TPU coated. **(B)** Elasticity of the anisotropic fabric. **(C)** Geometry of the sample for the property test and the fracture strength test of the heat-sealing seam. **(D)** Experimental configuration of material property test. **(E)** Experimental configuration of the fracture strength test.

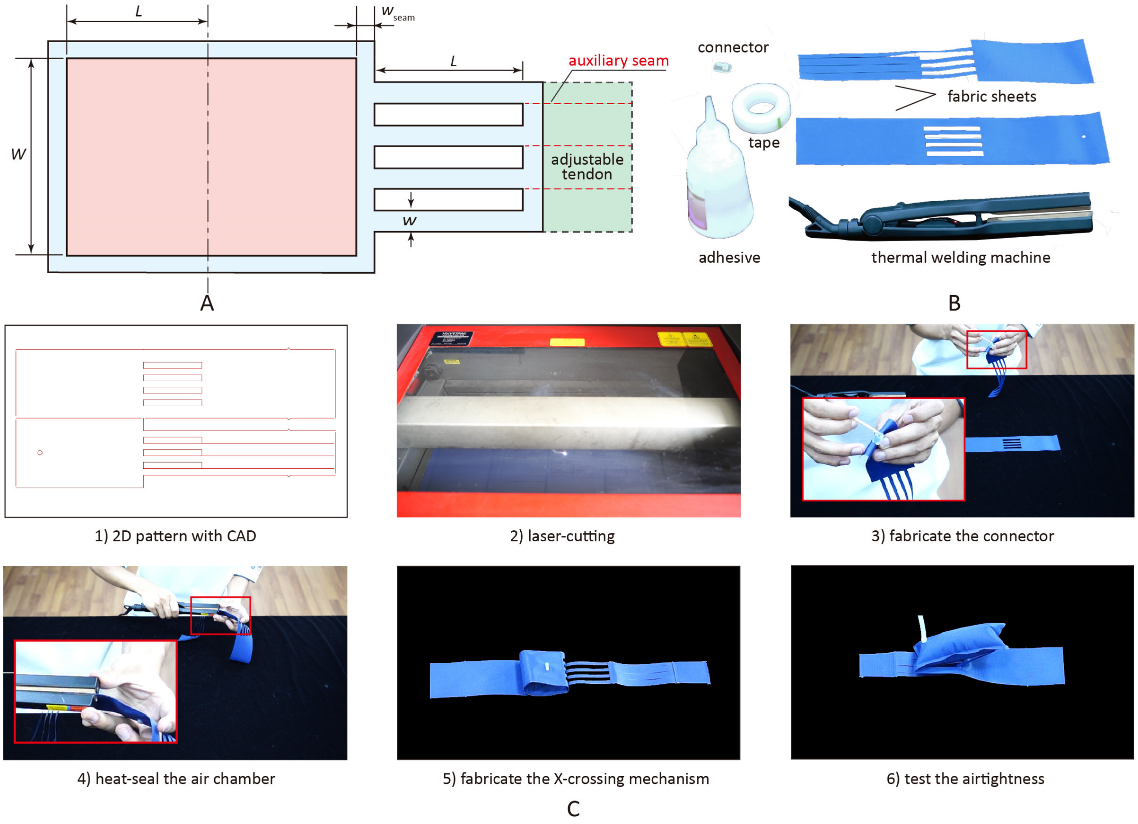

Fig. S2. Geometry of fabric sheet and fabrication of X-PAMs. (A) Geometry of the lower fabric sheet of X-PAMs (The upper sheet is shown in Fig. 2B). **(B)** Main components and tools to fabricate an X-PAM. **(C)** Fabrication procedures of X-PAMs (**Movie S1** and **2**): 1) Draw the 2D pattern of the fabric sheets with AutoCAD 2020 (AutoDesk, USA); 2) Use the laser cutter to obtain the fabric sheets; 3) Adhere the air connector on the upper sheet; 4) Heat-seal the air chamber; 5) Fabricate the X-crossing mechanism (including opening the filaments, crossing the filaments, and closing the filaments with a connecting piece); 6) Test the airtightness of the X-PAM.

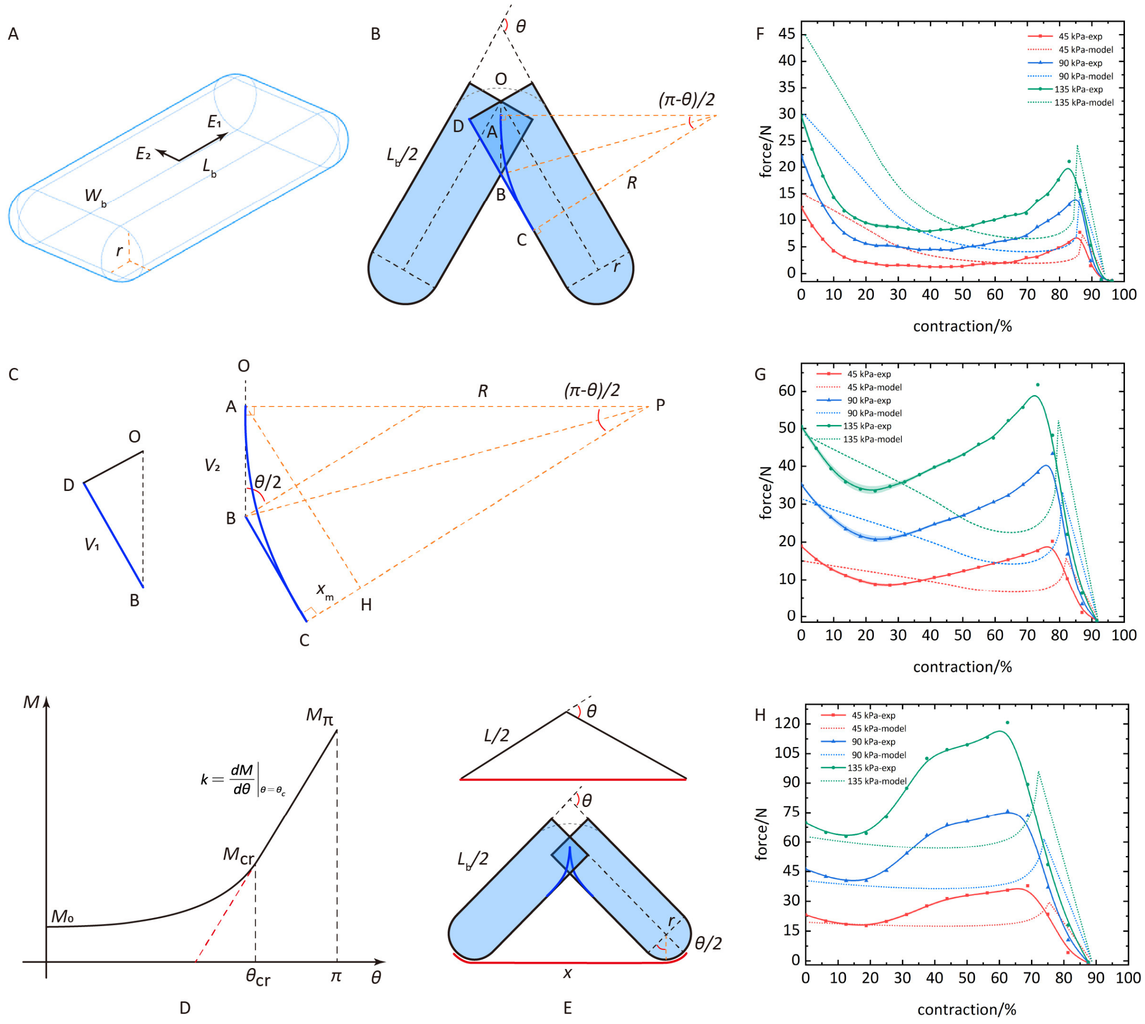

Fig. S3. Modeling of X-PAMs. (A) An X-PAM is approximated as a fillet box. **(B)** Geometry of an X-PAM with a bending angle of θ. **(C)** Detailed geometry of vanishing volume constitutes a triangle area (left) and a curved triangle area (right). **(D)** Diagram of expanding torque when bending the chamber of an X-PAM. When an X-PAM moves into the flatting stage $\theta \in [\theta_{cr}, \pi]$, the expanding torque is obtained by linear estimation. **(E)** Relationship between the end distance (red curve) and the bending angle θ. The upper diagram shows the configuration not considering the expansion of the chamber, and the lower diagram shows the configuration with the expansion radius considered. **(F)-(H)** Relationship between output force and contraction ratio.

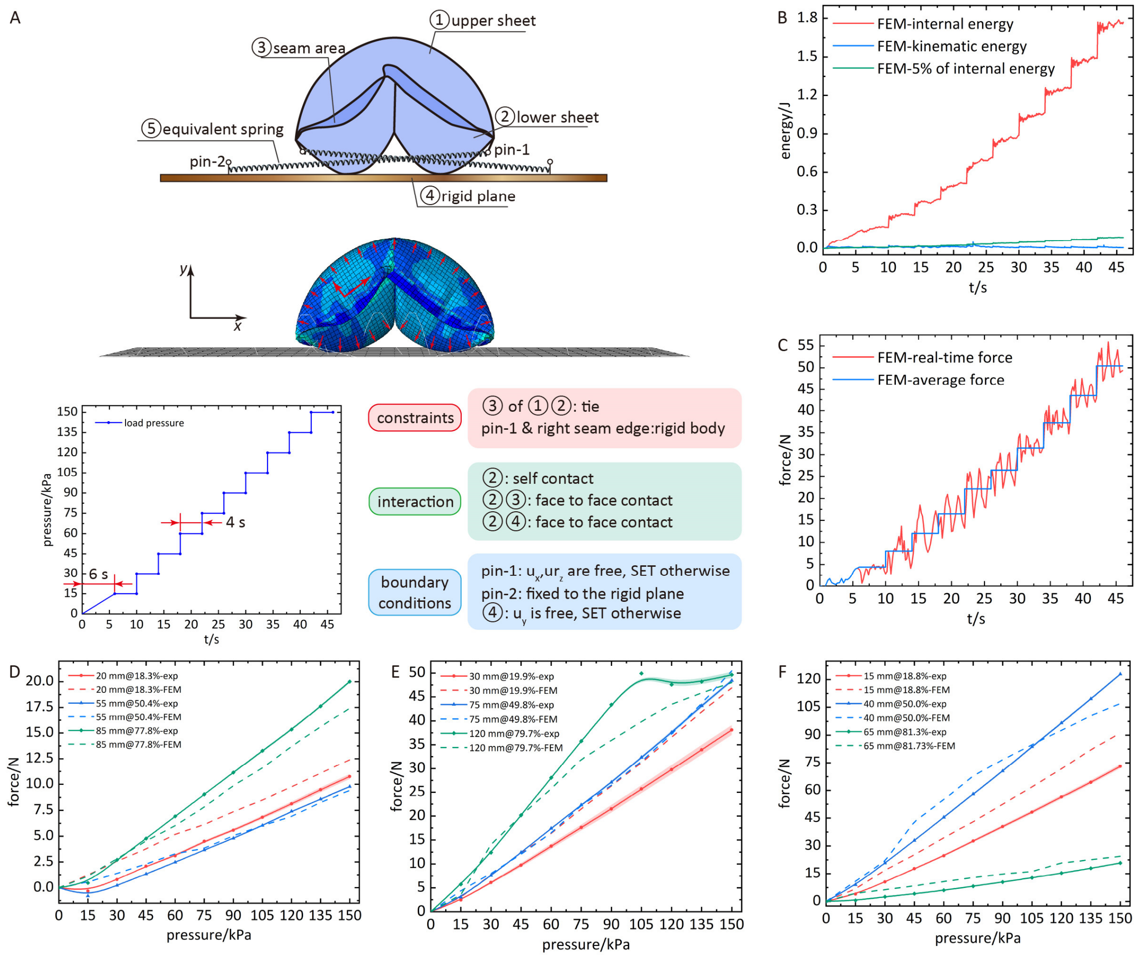

Fig. S4. Output force simulation of X-PAMs. (A) Simulation model and configurations of finite element method (FEM). The filaments and adjustable tendons are equivalent to a pair of springs to avoid complicated contacts and reduce simulation time. The constraints and boundary conditions are centrosymmetric. **(B)** The energy of the actuation process, where the kinematic energy is less than 5% of the internal energy. **(C)** The second half series (2 s) of the force data (4 s) under each pressure is used to calculate the average output force. **(D)** Simulation and experiment output force of the type 1 X-PAM (W/L=1:2). **(E)** Simulation and experiment output force of the type 2 X-PAM (W/L=1:1). **(F)** Simulation and experiment output force of the type 3 X-PAM (W/L=2:1). All these contraction ratios are presented according to the principle of proximity around 20%, 50% and 80%.

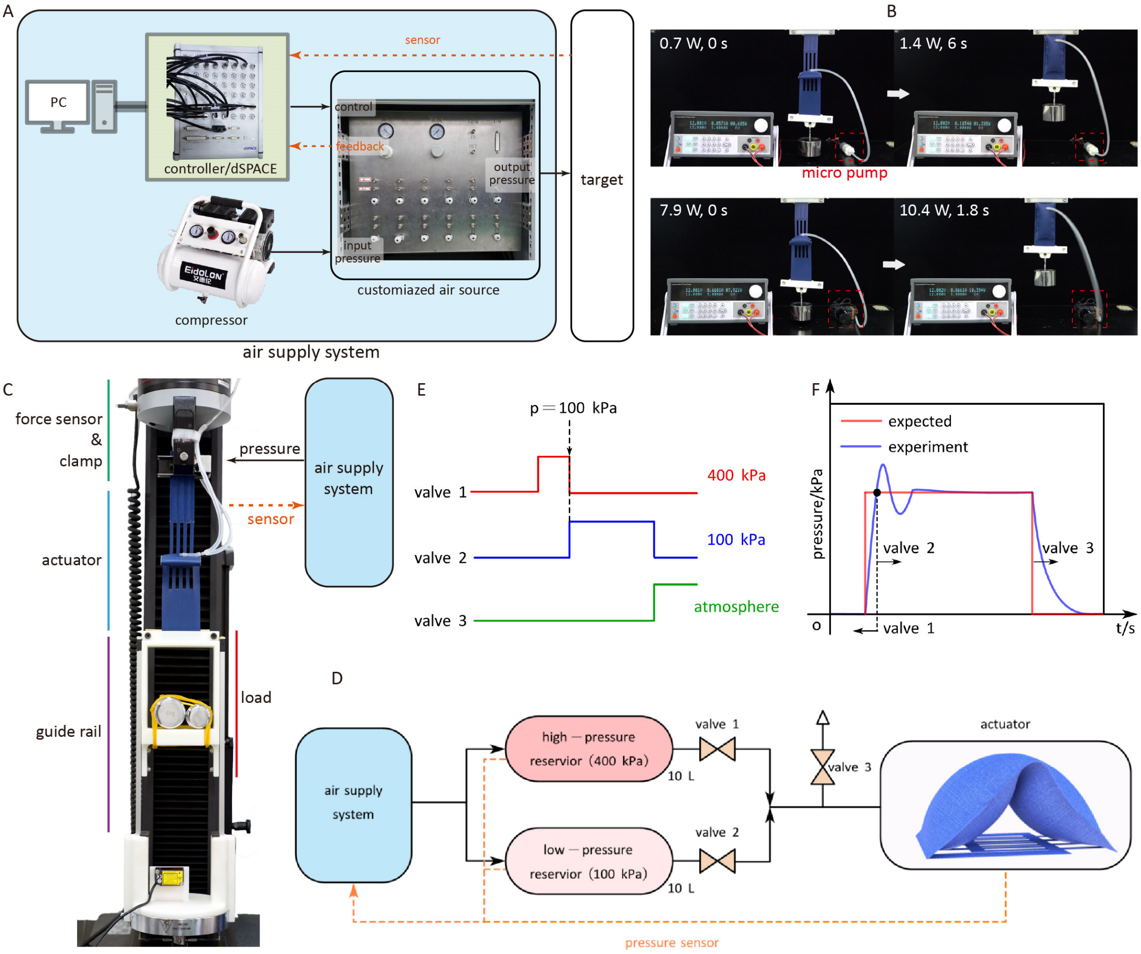

Fig. S5. Experimental setup of air supply. (A) Air supply system used in the experiments to obtain data. **(B)** Examples of portable deployment of air supply with micro pumps. **(C)** The experimental setup to fix the load. In these experiments, air pressure, airflow, displacement, and force are recorded. **(D)** Two air reservoirs are adopted to achieve rapid and accurate step stimuli of pressure. **(E)** Timings of valves for step stimuli. **(F)** Expected and experimental pressure of step stimuli.

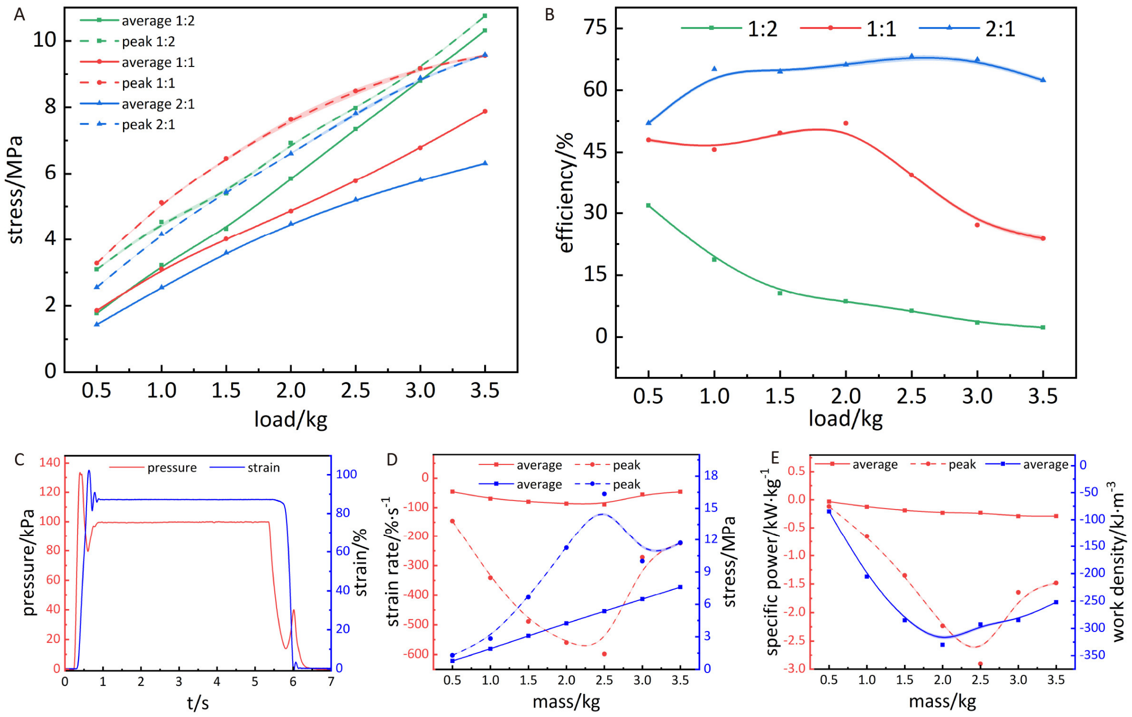

Fig. S6. Supplementary performance indicators. (A) Average and peak actuation stress of X-PAMs. **(B)** Efficiency of X-PAMs. **(C)** Pressure and strain of step response with the deflating process included (type 2 X-PAM with a load of 2 kg, connected to the atmosphere through the air tube and solenoid valve, also for D and E). There is a local bulge during the process of pressure drop because the decreasing rate of chamber volume exceeds the rate of air venting. **(D)** Strain rate and actuation stress of the deflating process. **(E)** Specific power and work density of the deflating process.

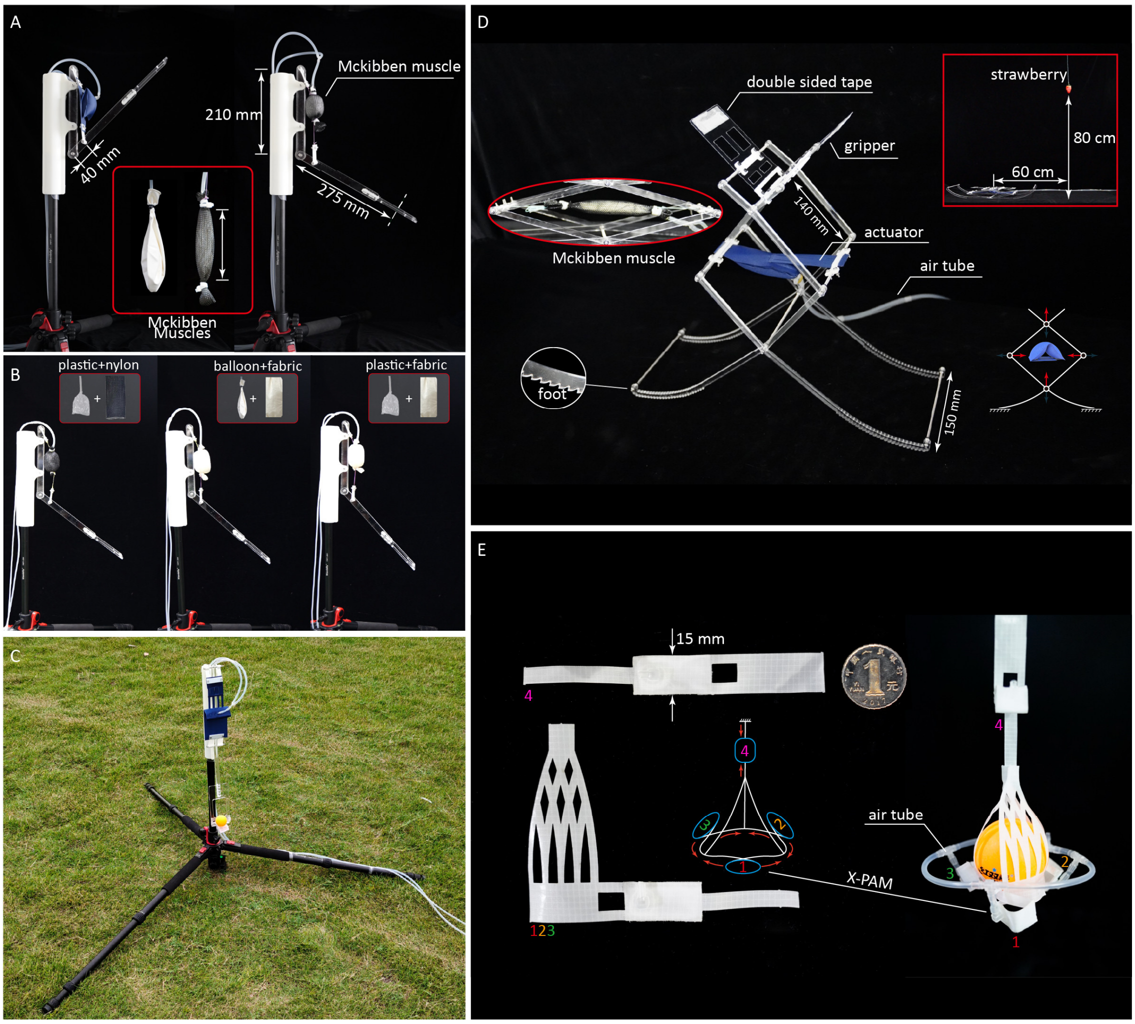

Fig. S7. Detailed configuration of the exemplary applications. (A) Configuration of the robotic elbow with X-PAM and a comparative McKibben muscle (balloon+nylon sheath). **(B)** Performance of the robotic elbow using McKibben muscles with different material combinations: plastic chamber+nylon sheath, balloon+ fabric sheath, and plastic chamber+ fabric sheath (left to right). Their flexion angles are close and below 60°. **(C)** Robotic elbow vertically throwing ping-pong ball in open areas. **(D)** Configuration of the jumping robot when actuated (standing up). **(E)** Configuration of the gripper.

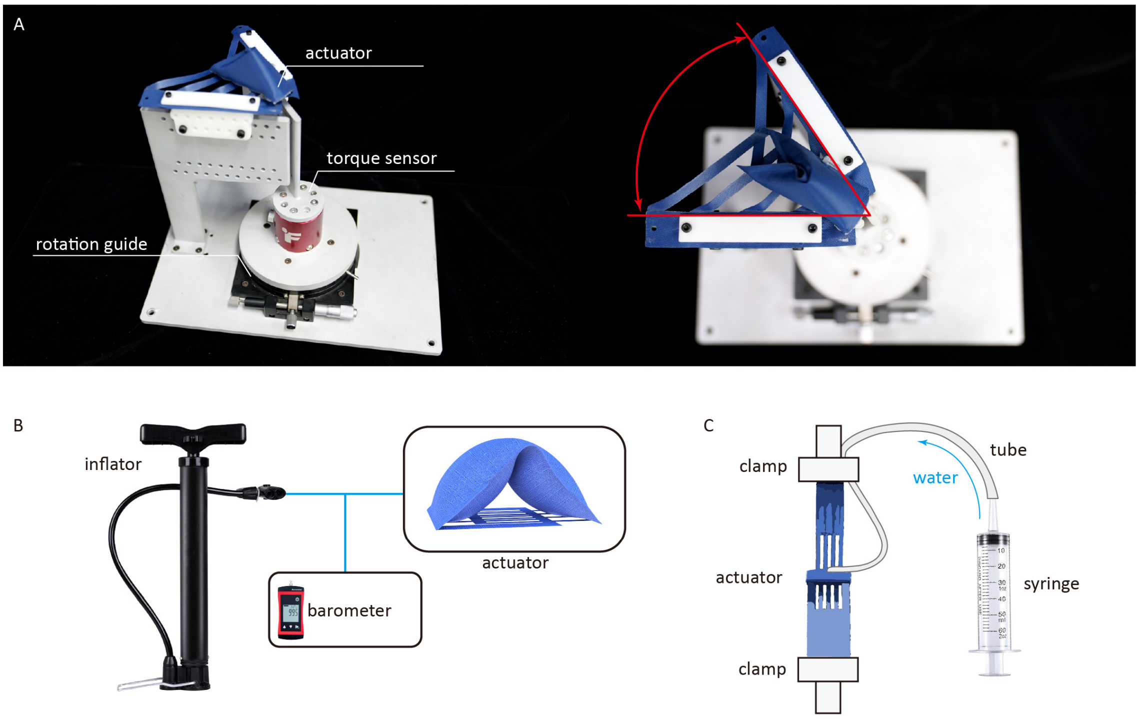

Fig. S8. Experimental setup for torque measurement and manual actuation devices. **(A)** Experimental setup for torque measurement, where the rotation guide can adjust and keep the angle of the actuator. **(B)** Manual device for pneumatic actuation. **(C)** Manual device for hydraulic actuation.

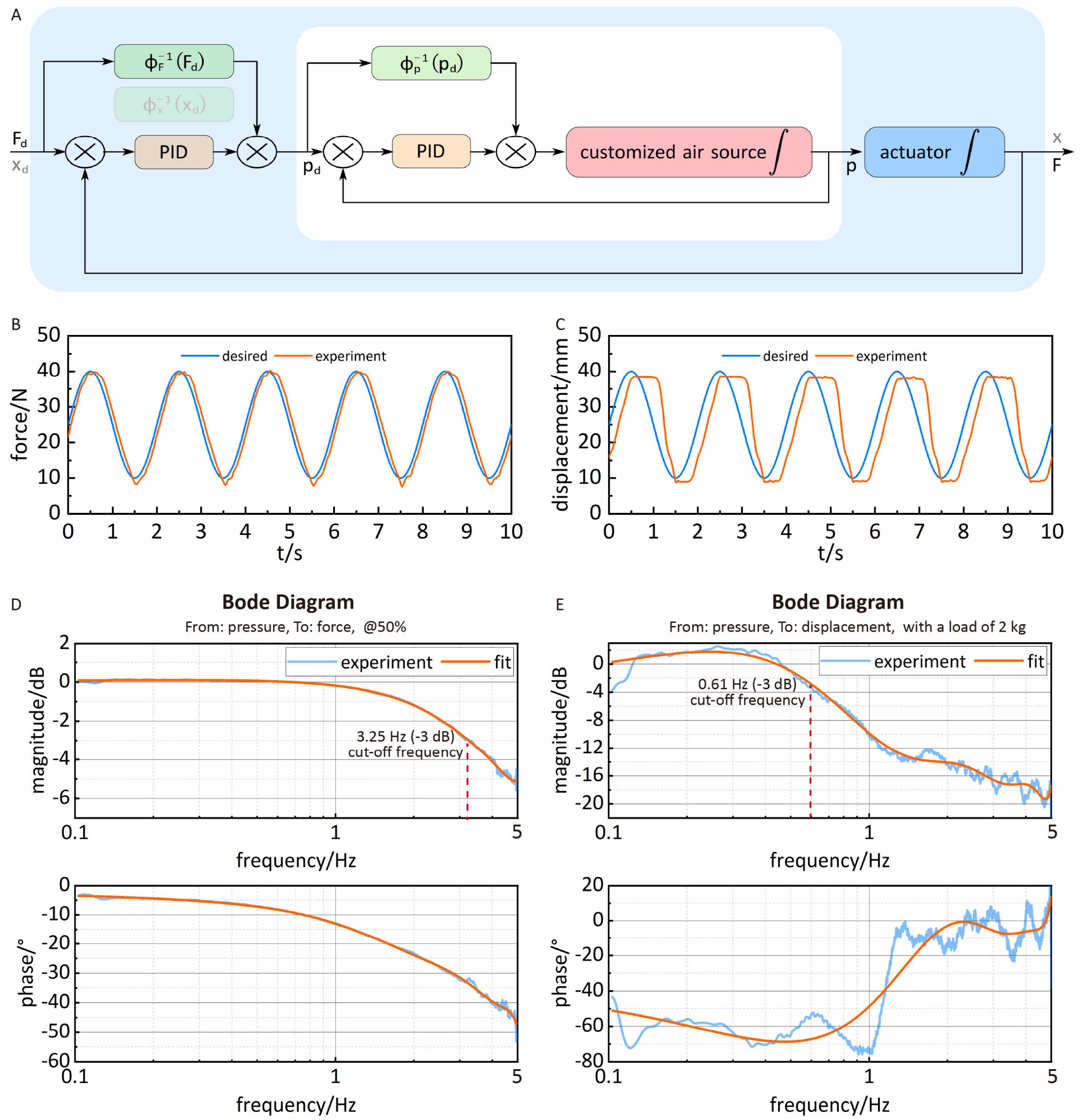

Fig. S9. Force/displacement tracking and frequency response experiments. (A) Diagram of control strategy in these tracking experiments. **(B)** Output force tracking: type 2 X-PAM, desired sinusoidal force: 10-40 N, frequency: 0.5 Hz, contraction ratio fixed: 50%. **(C)** Output displacement tracking: type 2 X-PAM, desired sinusoidal displacement: 10-40 mm, frequency: 0.5 Hz, load: 2 kg. **(D)** Frequency response of X-PAM: type 2, from pressure (0-100 kPa) to force, fixed at the contraction ratio of 50%, cut-off frequency 3.25 Hz (-3 dB). **(E)** Frequency response of X-PAM: type 2, from pressure (0-100 kPa) to displacement, with a load of 2 kg applied, cut-off frequency 0.61 Hz (-3 dB). Polynomials of degree six are used in D and E to fit the experimental curves to show the results more clearly.

Movie S1 to S9

Movie S1. Design and principle of X-PAMs

Movie S2. Fabrication of X-PAMs

Movie S3. Step response of X-PAMs

Movie S4. Force and displacement tracking

Movie S5. Robotic elbow

Movie S6. Jumping robot

Movie S7. Soft gripper with small-scale X-PAMs

Movie S8. Working in extreme environments

Movie S9. Mechanical extendibility of X-PAMs

Data S1: A collection of 3D models and FEM codes, including:

X-PAM type 1.dwg

X-PAM type 2.dwg

X-PAM type 3.dwg

small scale X-PAM.dwg

large scale X-PAM.dwg

ring-shaped X-PAM.dwg

asymmetric X-PAM.dwg

robotic elbow.dwg

jumping robot.dwg

soft gripper.dwg

PET for origami-combined X-PAM.dwg

FEM project.cae

FEM Python code.py

REFERENCES AND NOTES

1. I. W. Hunter, S. Lafontaine, paper presented at Technical Digest IEEE Solid-State Sensor and Actuator Workshop, Hilton Head Island, SC, 22 to 25 June 1992.

2. J. Madden, N. A. Vandesteeg, P. A. Anquetil, P. Madden, A. Takshi, R. Z. Pytel, S. R. Lafontaine, P. A. Wieringa, I. W. Hunter, Artificial muscle technology: Physical principles and naval prospects. *IEEE J. Oceanic Eng.* **29**, 706–728 (2004).

3. P. Rothemund, N. Kellaris, S. K. Mitchell, E. Acome, C. Keplinger, HASEL artificial muscles for a new generation of lifelike robots-recent progress and future opportunities. *Adv. Mater.* **33**, e2003375 (2021).

4. W. Liang, H. Liu, K. Wang, Z. Qian, L. Ren, L. Ren, Comparative study of robotic artificial actuators and biological muscle. *Adv. Mech. Eng.* **12**, 168781402093340 (2020).

5. J. Wang, D. Gao, P. S. Lee, Recent progress in artificial muscles for interactive soft robotics. *Adv. Mater.* **33**, e2003088 (2021).

6. J. Mohd Jani, M. Leary, A. Subic, M. A. Gibson, A review of shape memory alloy research, applications and opportunities. *Mater. Des.* **56**, 1078–1113 (2014).

7. Y. Wang, T. Nitta, Y. Hiratsuka, K. Morishima, In situ integrated microrobots driven by artificial muscles built from biomolecular motors. *Sci. Robot.* **7**, eaba8212 (2022).

8. Y. Zhao, C.-Y. Lo, L. Ruan, C.-H. Pi, C. Kim, Y. Alsaid, I. Frenkel, R. Rico, T.-C. Tsao, X. He, Somatosensory actuator based on stretchable conductive photothermally responsive hydrogel. *Sci. Robot.* **6**, eabd5483 (2021).

9. J. Foroughi, G. Spinks, Carbon nanotube and graphene fiber artificial muscles. *Nanoscale Adv.* **1**, 4592–4614 (2019).

10. H. Chu, X. Hu, Z. Wang, J. Mu, N. Li, X. Zhou, S. Fang, C. S. Haines, J. W. Park, S. Qin, N. Yuan, J. Xu, S. Tawfick, H. Kim, P. Conlin, M. Cho, K. Cho, J. Oh, S. Nielsen, K. A. Alberto, J.

M. Razal, J. Foroughi, G. M. Spinks, S. J. Kim, J. Ding, J. Leng, R. H. Baughman, Unipolar stroke, electroosmotic pump carbon nanotube yarn muscles. *Science* **371**, 494–498 (2021).

11. S. M. Mirvakili, I. W. Hunter, Artificial muscles: Mechanisms, applications, and challenges. *Adv. Mater.* **30**, e1704407 (2018).

12. J. Zhang, J. Sheng, C. T. O'Neill, C. J. Walsh, R. J. Wood, J.-H. Ryu, J. P. Desai, M. C. Yip, Robotic artificial muscles: Current progress and future perspectives. *IEEE Trans. Robot.* **35**, 761–781 (2019).

13. M. Taghavi, T. Helps, J. Rossiter, Electro-ribbon actuators and electro-origami robots. *Sci. Robot.* **3**, eaau9795 (2018).

14. M. Duduta, E. Hajiesmaili, H. Zhao, R. J. Wood, D. R. Clarke, Realizing the potential of dielectric elastomer artificial muscles. *Proc. Natl. Acad. Sci. U.S.A.* **116**, 2476–2481 (2019).

15. Z. Wang, K. Li, Q. He, S. Cai, A light-powered ultralight tensegrity robot with high deformability and load capacity. *Adv. Mater.* **31**, e1806849 (2018).

16. I. H. Kim, S. Choi, J. Lee, J. Jung, J. Yeo, J. T. Kim, S. Ryu, S.-K. Ahn, J. Kang, P. Poulin, S. O. Kim, Human-muscle-inspired single fibre actuator with reversible percolation. *Nat. Nanotechnol.* **17**, 1198–1205 (2022).

17. H. F. M. Ali, Y. Kim, Novel artificial muscle using shape memory alloy spring bundles in honeycomb architecture in Bi-directions. *Microsyst. Technol.* **28,** 2315–2324 (2022).

18. H. Zhang, S. Oh, M. Mahato, H. Yoo, I.-K. Oh, Knot-architectured fabric actuators based on shape memory fibers. *Adv. Funct. Mater.* **32**, 2205732 (2022).

19. A. Maziz, A. Concas, A. Khaldi, J. Stålhand, N.-K. Persson, E. W. H. Jager, Knitting and weaving artificial muscles. *Sci. Adv.* **3**, e1600327 (2017).

20. L. Hines, K. Petersen, G. Z. Lum, M. Sitti, Soft actuators for small-scale robotics. *Adv. Mater.* **29**, e1603483 (2017).

21. J. Mu, M. Jung de Andrade, S. Fang, X. Wang, E. Gao, N. Li, S. H. Kim, H. Wang, C. Hou, Q. Zhang, M. Zhu, D. Qian, H. Lu, D. Kongahage, S. Talebian, J. Foroughi, G. Spinks, H. Kim, T. H. Ware, H. J. Sim, D. Y. Lee, Y. Jang, S. J. Kim, R. H. Baughman, Sheath-run artificial muscles. *Science* **365**, 150–155 (2019).

22. C. S. Haines, M. D. Lima, N. Li, G. M. Spinks, J. Foroughi, J. D. W. Madden, S. H. Kim, S. Fang, M. Jung de Andrade, F. Göktepe, Ö. Göktepe, S. M. Mirvakili, S. Naficy, X. Lepró, J. Oh, M. E. Kozlov, S. J. Kim, X. Xu, B. J. Swedlove, G. G. Wallace, R. H. Baughman, Artificial muscles from fishing line and sewing thread. *Science* **343**, 868–872 (2014).

23. D. R. Higueras-Ruiz, M. W. Shafer, H. P. Feigenbaum, Cavatappi artificial muscles from drawing, twisting, and coiling polymer tubes. *Sci. Robot.* **6**, eabd5383 (2021).

24. S. Tawfick, Y. Tang, Stronger artificial muscles, with a twist. *Science* **365**, 125–126 (2019).

25. E. Acome, S. K. Mitchell, T. G. Morrissey, M. B. Emmett, C. Benjamin, M. King, M. Radakovitz, C. Keplinger, Hydraulically amplified self-healing electrostatic actuators with muscle-like performance. *Science* **359**, 61–65 (2018).

26. S. Li, D. M. Vogt, D. Rus, R. J. Wood, Fluid-driven origami-inspired artificial muscles. *Proc. Natl. Acad. Sci. U.S.A.* **114**, 13132–13137 (2017).

27. S. M. Mirvakili, D. Sim, I. W. Hunter, R. Langer, Actuation of untethered pneumatic artificial muscles and soft robots using magnetically induced liquid-to-gas phase transitions. *Sci. Robot.* **5**, eaaz4239 (2020).

28. M. Feng, D. Yang, C. Majidi, G. Gu, High-speed and low-energy actuation for pneumatic soft robots with internal exhaust air recirculation. *Adv. Intell. Syst.* **5**, 2200257 (2023).

29. N. Kellaris, V. Gopaluni Venkata, G. M. Smith, S. K. Mitchell, C. Keplinger, Peano-HASEL actuators: Muscle-mimetic, electrohydraulic transducers that linearly contract on activation. *Sci. Robot.* **3**, eaar3276 (2018).

30. G. Gu, J. Zou, R. Zhao, X. Zhao, X. Zhu, Soft wall-climbing robots. *Sci. Robot.* **3**, eaat2874 (2018).

31. G. M. Spinks, N. D. Martino, S. Naficy, D. J. Shepherd, J. Foroughi, Dual high-stroke and high-work capacity artificial muscles inspired by DNA supercoiling. *Sci. Robot.* **6**, eabf4788 (2021).

32. M. C. Huber, U. Jonas, S. M. Schiller, An autonomous chemically fueled artificial protein muscle. *Adv. Intell. Syst.* **4**, 2100189 (2022).

33. M. Kanik, S. Orguc, G. Varnavides, J. Kim, T. Benavides, D. Gonzalez, T. Akintilo, C. C. Tasan, A. P. Chandrakasan, Y. Fink, P. Anikeeva, Strain-programmable fiber-based artificial muscle. *Science* **365**, 145–150 (2019).

34. C.-P. Chou, B. Hannaford, paper presented at Proceedings of the 1994 IEEE International Conference on Robotics and Automation, San Diego, CA, 8 to 13 May 1994.

35. C. de Pascali, G. A. Naselli, S. Palagi, R. B. N. Scharff, B. Mazzolai, 3D-printed biomimetic artificial muscles using soft actuators that contract and elongate. *Sci. Robot.* **7**, eabn4155 (2022).

36. J.-G. Lee, H. Rodrigue, Origami-based vacuum pneumatic artificial muscles with large contraction ratios. *Soft Robot.* **6**, 109–117 (2019).

37. B. Tondu, P. Lopez, Modeling and control of McKibben artificial muscle robot actuators. *IEEE Control Syst. Mag.* **20**, 15–38 (2000).

38. H. D. Yang, B. T. Greczek, A. T. Asbeck, Modeling and analysis of a high-displacement pneumatic artificial muscle with integrated sensing. *Front. Robot. AI* **5**, 356 (2019).

39. S. Wang, E. F. Miranda, L. H. Blumenschein, The folded pneumatic artificial muscle (foldPAM): Towards programmability and control via end geometry. *IEEE Robot. Autom. Lett.* **8**, 1383–1390 (2023).

40. D. Bruder, R. J. Wood, The chain-link actuator: Exploiting the bending stiffness of McKibben artificial muscles to achieve larger contraction ratios. *IEEE Robot. Autom. Lett.* **7**, 542–548 (2022).

41. K. Han, N.-H. Kim, D. Shin, A novel soft pneumatic artificial muscle with high-contraction ratio. *Soft Robot.* **5**, 554–566 (2018).

42. J. Kwon, S. J. Yoon, Y.-L. Park, Flat inflatable artificial muscles with large stroke and adjustable force–Length relations. *IEEE Trans. Robot.* **36**, 743–756 (2020).

43. J. Liu, Z. Ma, Y. Wang, S. Zuo, Reconfigurable self-sensing pneumatic artificial muscle with locking ability based on modular multi-chamber soft actuator. *IEEE Robot. Autom. Lett.* **7**, 8635–8642 (2022).

44. M. Feng, D. Yang, G. Gu, High-force fabric-based pneumatic actuators with asymmetric chambers and interference-reinforced structure for soft wearable assistive gloves. *IEEE Robot. Autom. Lett.* **6**, 3105–3111 (2021).

45. A. Coutinho, J. H. Park, B. Jamil, H. R. Choi, H. Rodrigue, Hyperbaric vacuum-based artificial muscles for high-performance actuation. *Adv. Intell. Syst.* **5**, 2200090 (2023).

46. S. K. Mitchell, X. Wang, E. Acome, T. Martin, K. Ly, N. Kellaris, V. G. Venkata, C. Keplinger, An easy-to-implement toolkit to create versatile and high-performance HASEL actuators for untethered soft robots. *Adv. Sci.* **6**, 1900178 (2019).

47. C. Laschi, B. Mazzolai, M. Cianchetti, Soft robotics: Technologies and systems pushing the boundaries of robot abilities. *Sci. Robot.* **1**, eaah3690 (2016).

48. H. E. Huxley, The mechanism of muscular contraction. *Science* **164**, 1356–1366 (1969).

49. T. A. Duke, Molecular model of muscle contraction. *Proc. Natl. Acad. Sci. U.S.A.* **96**, 2770–2775 (1999).

50. G. K. Klute, B. Hannaford, paper presented at Proceedings 1998 IEEE/RSJ International Conference on Intelligent Robots and Systems. Innovations in Theory, Practice and Applications, Victoria, BC, 13 to 17 October 1998.

51. C.-P. Chou, B. Hannaford, Measurement and modeling of McKibben pneumatic artificial muscles. *IEEE Trans. Robot. Autom.* **12**, 90–102 (1996).

52. J. M. Winters, S.L.-Y. Woo, *Multiple Muscle Systems, Biomechanics and Movement Organization* (Springer, 1990).

53. W. Kim, H. Park, J. Kim, Compact flat fabric pneumatic artificial muscle (ffPAM) for soft wearable robotic devices. *IEEE Robot. Autom. Lett.* **6**, 2603–2610 (2021).

54. D. Xie, S. Zuo, J. Liu, A novel flat modular pneumatic artificial muscle. *Smart Mater. Struct.* **29**, 065013 (2020).

55. Z. Zhang, W. Fan, G. Chen, J. Luo, Q. Lu, H. Wang, paper presented at 2021 IEEE 4th International Conference on Soft Robotics (RoboSoft), New Haven, CT, 12 to 16 April 1992.

56. X. Wang, S. K. Mitchell, E. H. Rumley, P. Rothemund, C. Keplinger, High-strain Peano-HASEL actuators. *Adv. Funct. Mater.* **30**, 1908821 (2020).

57. M. D. Lima, N. Li, M. Jung de Andrade, S. Fang, J. Oh, G. M. Spinks, M. E. Kozlov, C. S. Haines, D. Suh, J. Foroughi, S. J. Kim, Y. Chen, T. Ware, M. K. Shin, L. D. Machado, A. F. Fonseca, J. D. W. Madden, W. E. Voit, D. S. Galvão, R. H. Baughman, Electrically, chemically, and photonically powered torsional and tensile actuation of hybrid carbon nanotube yarn muscles. *Science* **338**, 928–932 (2012).

58. J. A. Lee, N. Li, C. S. Haines, K. J. Kim, X. Lepró, R. Ovalle-Robles, S. J. Kim, R. H. Baughman, Electrochemically powered, energy-conserving carbon nanotube artificial muscles. *Adv. Mater.* **29**, e1700870 (2017).

59. R. Pelrine, R. Kornbluh, Q. Pei, J. Joseph, High-speed electrically actuated elastomers with strain greater than 100%. *Science* **287**, 836–839 (2000).

60. M. P. M. Dicker, A. B. Baker, R. J. Iredale, S. Naficy, I. P. Bond, C. F. J. Faul, J. M. Rossiter, G. M. Spinks, P. M. Weaver, Light-triggered soft artificial muscles: Molecular-level amplification of actuation control signals. *Sci. Rep.* **7**, 9197 (2017).

61. K. Choi, S. J. Park, M. Won, C. H. Park, Soft fabric muscle based on thin diameter SMA springs. *Smart Mater. Struct.* **31**, 055020 (2022).

62. J. Foroughi, G. M. Spinks, S. Aziz, A. Mirabedini, A. Jeiranikhameneh, G. G. Wallace, M. E. Kozlov, R. H. Baughman, Knitted carbon-nanotube-sheath/spandex-core elastomeric yarns for artificial muscles and strain sensing. *ACS Nano* **10**, 9129–9135 (2016).

63. Q. He, Z. Wang, Z. Song, S. Cai, Bioinspired design of vascular artificial muscle. *Adv. Mater. Technol.* **4**, 1800244 (2019).

64. L. Zhao, H. Tian, H. Liu, W. Zhang, F. Zhao, X. Song, J. Shao, Bio-inspired soft-rigid hybrid smart artificial muscle based on liquid crystal elastomer and helical metal wire. *Small* **19**, e2206342 (2023).

65. Q. He, Z. Wang, Y. Wang, A. Minori, M. T. Tolley, S. Cai, Electrically controlled liquid crystal elastomer-based soft tubular actuator with multimodal actuation. *Sci. Adv.* **5**, eaax5746 (2019).

66. R. Glaser, V. Caccese, Experimental methods to determine in-plane material properties of polyurethane-coated nylon fabric. *J. Text. Inst.* **104**, 682–698 (2013).

67. C. T. O'Neill, C. M. McCann, C. J. Hohimer, K. Bertoldi, C. J. Walsh, Unfolding textile-based pneumatic actuators for wearable applications. *Soft Robot.* **9**, 163–172 (2022).

68. P. H. Nguyen, W. Zhang, Design and computational modeling of fabric soft pneumatic actuators for wearable assistive devices. *Sci. Rep.* **10**, 9638 (2020).

69. L. Ge, F. Chen, D. Wang, Y. Zhang, D. Han, T. Wang, G. Gu, Design, modeling, and evaluation of fabric-based pneumatic actuators for soft wearable assistive gloves. *Soft Robot.* **7**, 583–596 (2020).

70. F. A. Morrison, “Data correlation for drag coefficient for sphere,” thesis Michigan Technological University, Houghton, MI (2016).
